# Elaboration on the architecture of pH-sensitive surface charge-adaptive micelles with enhanced penetration and bactericidal activity in biofilms

**DOI:** 10.1186/s12951-021-00980-8

**Published:** 2021-08-06

**Authors:** Rong Guo, Keke Li, Baocheng Tian, Changrong Wang, Xiangjun Chen, Xinyu Jiang, Huayu He, Wei Hong

**Affiliations:** School of Pharmacy, Shandong New Drug Loading and Release Technology and Preparation Engineering Laboratory, Binzhou Medical University, 346 Guanhai Road, Yantai, 264003 People’s Republic of China

**Keywords:** Biofilm targeting, pH-sensitive copolymers, Distribution of pH-sensitive segments, Implant-related biofilm infection

## Abstract

**Background:**

Biofilm formation is one of the main reasons for persistent bacterial infections. Recently, pH-sensitive copolymers have fascinated incredible attention to tackle biofilm-related infections. However, the proper incorporation of pH-sensitive segments in the polymer chains, which could significantly affect the biofilms targeting ability, has not been particularly investigated. Herein, we synthesized three types of pH-sensitive copolymers based on poly (β-amino ester) (PAE), poly (lactic-co-glycolic acid) (PLA) and polyethylene glycol (PEG), PAE-PLA-mPEG (A-L-E), PLA-PAE-mPEG (L-A-E) and PLA-PEG-PAE (L-E-A) to address this issue.

**Results:**

The three copolymers could self-assemble into micelles (M_A-L-E_, M_L-A-E_ and M_L-E-A_) in aqueous medium. Compared with M_A-L-E_ and M_L-A-E_, placing the PAE at the distal PEG end of PLA-PEG to yield PLA-PEG-PAE (M_L-E-A_) was characterized with proper triggering pH, fully biofilm penetration, and high cell membrane binding affinity. Further loaded with Triclosan (TCS), M_L-E-A_/TCS could efficiently kill the bacteria either in planktonic or biofilm mode. We reasoned that PAE segments would be preferentially placed near the surface and distant from the hydrophobic PLA segments. This would increase the magnitude of surface charge-switching capability, as the cationic PAE^+^ would easily disassociate from the inner core without conquering the additional hydrophobic force arising from covalent linkage with PLA segments, and rapidly rise to the outermost layer of the micellar surface due to the relative hydrophilicity. This was significant in that it could enable the micelles immediately change its surface charge where localized acidity occurred, and efficiently bind themselves to the bacterial surface where they became hydrolyzed by bacterial lipases to stimulate release of encapsulated TCS even a relatively short residence time to prevent rapid wash-out. I*n vivo* therapeutic performance of M_L-E-A_/TCS was evaluated on a classical biofilm infection model, implant-related biofilm infection. The result suggested that M_L-E-A_/TCS was effective for the treatment of implant-related biofilm infection, which was proved by the efficient clearance of biofilm-contaminated catheters and the recovery of surrounding infected tissues.

**Conclusions:**

In summary, elaboration on the architecture of pH-sensitive copolymers was the first step to target biofilm. The M_L-E-A_ structure may represent an interesting future direction in the treatment of biofilm-relevant infections associated with acidity.

**Graphic abstract:**

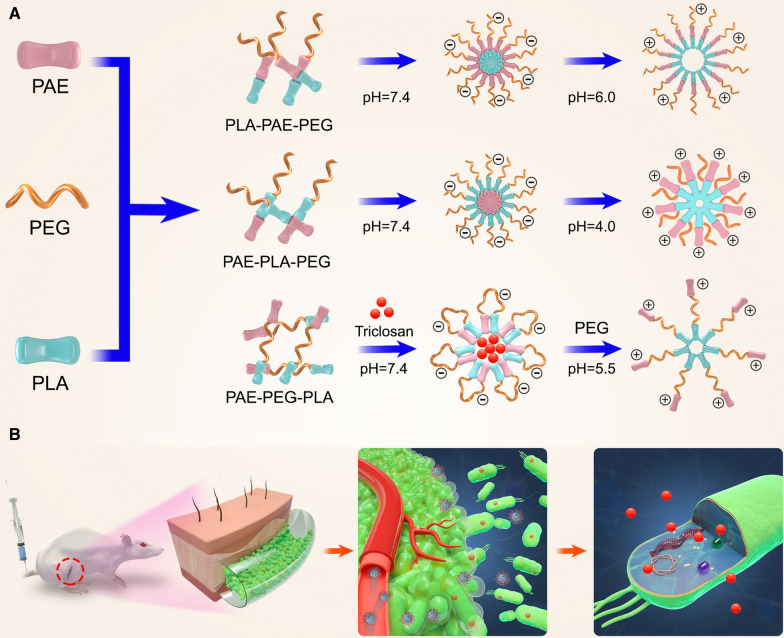

**Supplementary Information:**

The online version contains supplementary material available at 10.1186/s12951-021-00980-8.

## Introduction

Biofilm accounts for more than 60% of human infections of microorganisms [1–4]. Shielded by the protective matrix of the extracellular polymeric substances (EPS) produced by the biofilms [3, 5], the microorganisms can withstand up to 1000 times effective dose of antimicrobial agents compared with planktonic microorganisms [6]. To target biofilms-associated infections, treatments have been proposed in a variety of ways, such as inhibition and disruption of biofilm formation, improvement of the penetration ability of antimicrobial agents into the EPS matrix and so on [7].

In recent years, design of micelles has enabled potential strategies to overcome the resistance of biofilms to antibacterial drugs [8–11]. However, studies have shown that the anti-biofilm efficacy of plain micelles was greatly limited due to the lack of penetration and retention ability [12]. Cationic micelles have since been conducted to increase the electrostatic interaction with negative component in biofilm, and enhanced in vitro anti-biofilm activity has been observed [13–15]. Unfortunately, cationic micelles have a short half-life in vivo and may cause potential toxicity through non-specific binding [16]. To overcome these shortcomings, pH-sensitive copolymers are introduced to construct pH-sensitive surface charge-adaptive micelles (SCAMs), which have been proposed in the treatment of biofilm associated infections [17, 18]. It is well documented that sugar fermentation creates a relatively acidic environment for the biofilm [19, 20], which triggers the conversion of SCAMs from neutral at the physiological pH of 7.4 to cationic. Hence, the infection specific targeting can be achieved while non-specific interactions are minimized [21–23]. Shi et al. have developed SCAMs composed of two copolymers, poly(ε-caprolactone)-b-poly (β-amino ester) (PCL-b-PAE) and poly(ethylene glycol)-b-poly (ε-caprolactone) (PEG-b-PCL) [24]. The micelle surface becomes hydrophilic and positive at acidic conditions while remaining hydrophobic and negative at the physiological pH, which enabled the SCAMs to permeate through and function in the biofilms. Another SCAMs based on poly(D,L-lactic-co-glycolic acid)-b-poly (L-histidine)-b-poly-(ethylene glycol) (PLGA-PLH-PEG) was developed by Farokhzad et al. to enhance biofilm penetration [25]. They reasoned that by placing the PLH between the PLGA and PEG to yield the linear structure of PLGA-PLH-PEG could not only maximize the surface charge-switching capability at acidic pH but also improve micelles colloidal stability and circulation time at physiological pH. These results indicated that how to incorporate the pH-sensitive segments in the polymer chains greatly affected the charge-switching capability. Therefore, the architecture of pH-sensitive copolymers should be rationally designed before using, which has been generally ignored in most studies.

Usually, a typical pH-sensitive copolymer consists of three types of segments, hydrophobic segments, hydrophilic segments and pH-sensitive segments. The hydrophobic segments could form a solid inner core where drugs are loaded. The hydrophilic segments could enable the micelles escape from the rapid clearance by the mononuclear phagocytic system [12]. The pH-sensitive segments are responsible for switching the surface charge when the pH changes to acidic at the infection site [17]. It is clear that essential for biofilm penetration are both stealth in the blood and avid bacterial binding upon arriving at the acidity-associated infection. Poly (ethylene glycol) (PEG) has been amply applied to provide stealth properties to materials, making them “invisible” to cells, as well as resistant to protein adsorption. However, a big disadvantage induced by the PEGylation, called “PEG dilemma” phenomenon, was that PEG also hampered the electrostatic interaction between cationic micelles and bacteria. Additionally, in order to maximize the surface charge-switching capability, the pH-sensitive segments would be preferentially placed near the micelles surface.

**Fig. 1:**
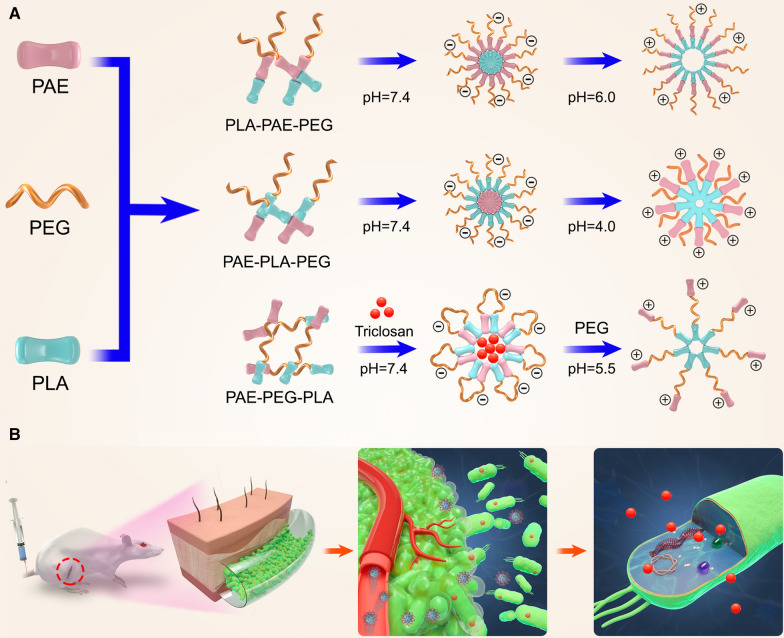
Elaboration on the architecture of pH-sensitive copolymers with proper pH_t_ (**a**). The mechanism for targeting delivery of encapsulated drug into bacteria deep into biofilms using SCAMs for treatment of implant-related biofilm infections (**b**)

Herein, our interests focused on elaborating the pH-sensitive copolymers to minimize the nontarget interactions at physiologic pH 7.4 and produce strong multivalent electrostatic-mediated binding at acidic pH for biofilm treatment. We prepared three types of pH-sensitive copolymers with similar compositions but different distributions of pH-sensitive segments in the chains, PLA-PAE-mPEG, PAE-PLA-mPEG and PLA-PEG-PAE. As shown in Fig. 1, three copolymers could self-assembly into micelles M_L-A-E_, M_A-L-E_ and M_L-E-A_ with PEG as the stable shell, PLA as the hydrophobic core and PAE as the pH-sensitive hydrophobic core moieties. The triggering pH (the pH at which a remarkable surface charge reversal occurs, pH_t_) was firstly investigated. It is noteworthy that, both M_L-A-E_ and M_L-E-A_ could elaborately control charge switching under the biofilm environment, with the pH_t_ of 5.5 and 6.0, respectively. On the contrary, the pH_t_ of M_A-L-E_ was 4.0, which was much lower than biofilm environmental pH (≈ 5.5), and not suitable for targeting biofilm. Next, the pH-dependent physicochemical properties of M_L-A-E_ and M_L-E-A_ (including particle size and zeta potential, micelles-bacteria binding affinity, and biofilm penetration) were characterized. Further loaded with Triclosan (TCS), the antibacterial efficacy and biofilm eradication of M_L-A-E_/TCS and M_L-E-A_/TCS were also evaluated in vitro and in vivo to reveal the structure–function relationship of pH-sensitive copolymers. Results demonstrated that M_L-E-A_ promoted the effective penetration and long-term retention inside biofilms, and were more effective in killing bacteria deep into biofilm both in vitro and in vivo compared to free TCS or TCS encapsulated in M_L-A-E_. This study may provide significant suggestion in designing pH-sensitive copolymers for biofilm targeting.

## Materials and methods

### Materials

#### Reagents

The copolymers of PLA_5K_-PAE_5K_-mPEG_5K_, PAE_5K_-PLA_5K_-mPEG_5K_ and PLA_5K_-PEG_5K_-PAE_5K_ were purchased from Ruixi Biological Technology Co., Ltd (Xi’an, Shanxi, China). The details of synthesis and characterization of the copolymers were shown in the Additional file 1. Biotin LPS and A LIVE/DEAD^®^ *Bac*Light™ Bacterial Viability Kit were obtained from Nanocs, Inc. (New York, USA) and Thermo Fisher Scientific Inc. (Shanghai, China), respectively. Nile red and Triclosan (TCS) were obtained from Macklin Biochemical Co., Ltd (Shanghai, China).

#### Bacteria

Two bacteria strains, *Staphylococcus aureus* ATCC 29213 and *Escherichia coli* ATCC 25922, were obtained from American Type Culture Collection (VA, USA), which were maintained in 30% glycerol at – 80 ºC until use.

#### Animals

Male Sprague–Dawley rats (220–250 g) were subject to the in vivo treatment of implant-related biofilm infection. The SD rats were obtained from Pengyue Laboratory Animal Breeding Co., Ltd (Jinan, Shandong, China) and the animal studies were conducted according to the experimental protocols by Institutional Animal Care and Use Committee of Binzhou Medical University.

#### Micellar formulations

M_L-E-A_: blank pH-sensitive surface charge-adaptive micelles composed of PLA-PEG-PAE;

M_L-A-E_: blank pH-sensitive surface charge-adaptive micelles composed of PLA-PAE-mPEG;

M_A-L-E_: blank pH-sensitive surface charge-adaptive micelles composed of PAE-PLA-mPEG;

M_L-E-A_/TCS: M_L-E-A_ loaded with TCS;

M_L-A-E_/TCS: M_L-A-E_ loaded with TCS;

M_L-E-A_/Nile Red: M_L-E-A_ loaded with Nile Red;

M_L-A-E_/Nile Red: M_L-A-E_ loaded with Nile Red.

### Methods

#### Triggering pH (pH_t_) analysis

M_L-A-E_, M_L-E-A_ and M_A-L-E_ were fabricated by the thin-film hydration method [26]. Briefly, 50 mg of PLA-PAE-mPEG, PLA-PEG-PAE and PAE-PLA-mPEG were dissolved in 20 ml of dichloromethane, respectively. Then, the solvent was removed by rotary evaporation to allow the film forming. After that, the film was hydrated with 20 mL of PBS (pH 7.4), and filtrated through a 0.22 μm film to obtain micellar solution. Finally, the pH of M_L-E-A_, M_L-A-E_ and M_A-L-E_ was adjusted to 3.0, 4.0, 4.5, 5.0. 5.5 and 6.0, respectively. The zeta potential of each micellar preparation was measured on a Zetasizer Nano ZS analyzer (DLS, Malvern, UK).

#### pH-dependent physicochemical properties

The changes of morphology, particle size and zeta potential of M_L-E-A_ and M_L-A-E_ under pH 7.4 and 5.5 over time were investigated on a JEM1400 transmission electron microscopy (TEM, JEOL, Japan) and a Zetasizer Nano ZS analyzer (DLS, Malvern, UK), respectively.

#### Micelles-bacterium binding studies

##### Zeta potential analysis

The planktonic bacteria-micelle binding study was initialized by adding 10 mL of the tested blank micelles (M_L-E-A_ and M_L-A-E_) to 10 mL of bacterial suspensions (10^8^ CFU) with pH values adjusted to 5.5 and 7.4, respectively. The zeta potential of the micelles/bacteria mixture was measured on a Zetasizer Nano ZS analyzer (Malvern, UK) for each solution with time points ranging from 0 to 24 h.

##### Confocal laser scanning microscope (CLSM)

The bacteria were suspended in PBS at pH of 5.5 or 7.4, and incubated with M_L-E-A_/Nile Red or M_L-A-E_/Nile Red solutions, respectively. As scheduled time points (1, 2, 4 and 8 h), the unbound micelles were removed by rinsing the bacteria solution twice with saline, and the bacteria were resuspended in 100 μL PBS at pH 7.4. The red fluorescence was measured at λex (583 nm)/λem (688 nm) to obtain the microscope images by a Leica TCS SPE Microsystems (Wetzlar, Germany). Fiji Image J software was used to measure the relative red fluorescence intensity.

##### Flow cytometry assays

The red fluorescence data of the bacterial suspensions prepared in the CLSM section was also acquired by BD FACSCanto II flow cytometry (USA). The fluorescence intensity of the cells was calculated by means of the histogram plot with the untreated negative sample as control.

##### Bio-layer interferometry (BLI)

The BLI study was performed on Octet RED 96e (ForteBio, USA). The pH of M_L-E-A_ and M_L-A-E_ was firstly adjusted to 7.4 and 5.5, respectively. The biotin-linked lipopolysaccharide (b-LPS) was loaded on streptavidin (SA) biosensor. Association and dissociation experiments were conducted for 90 and 120 s, respectively.

#### pH-dependent biofilm penetration

2 mL of bacteria was added into petri dishes and cultured for 4 d to establish mature biofilms. Then, the petri dishes with mature biofilms attached were incubated with M_L-E-A_/Nile Red and M_L-A-E_/Nile Red for 1 h, 2 h and 4 h under pH 7.4 and 5.5, respectively. The biofilm images were obtained on a Zeiss LSM 880 (Zeiss, Germany) after stained with SYTO 9 for 30 min. The Z-stack imaging was carried out using the areas near the center of the dishes at a 1-μm interval.

#### Preparation and characterization of TCS-loaded micelles

The M_L-E-A_/TCS and M_L-A-E_/TCS were also prepared by thin-film hydration method with 10 mg of TCS loaded. The morphology of the micelles was investigated on a JEM1400 TEM (JEOL, Japan), and the particle size and zeta potential were measured on a Zetasizer Nano ZS (Malvern, UK). Drug loading coefficient (DL%) was calculated by Eq. (1):

1 $$DL\%=\frac{\mathrm{Weight\,of\,the\, drug\, in\, micelles}}{\mathrm{Weight\, of\, the\, feeding\, copolymer\, and\, drug}}\times 100\%,$$

The entrapment efficiency (EE%) was calculated by Eq. (2):

2 $$EE\%=\frac{\mathrm{Weight\, of\, the\, drug\, in\, micelles}}{\mathrm{Weight\, of\, the\, feeding\, drug}}\times 100\%.$$

A dialysis bag (WM, 12–14 kDa) containing 2 mL freshly prepared M_L-E-A_/TCS and M_L-A-E_/TCS was incubated in 20 mL PBS (10 mM, pH 5.5 or pH 7.4), and aliquots of the dialysis solution were collected at predetermined time intervals, which was subject to absorbance measurement at 281 nm on a Synergy H1 microplate reader (Biotek Instruments, Inc., USA). The cumulative drug release *vs* time was plotted. The Lipase-triggered release behavior of TCS was studied after adding Lipase to PBS (10 mM, pH 5.5 or pH 7.4) with a final concentration of 0.5 mg/mL as described above.

#### In vitro antibacterial activity against planktonic bacteria

##### Minimum inhibitory concentration

The minimal inhibitory concentrations (MICs) of free TCS or TCS-loaded micelles (M_L-E-A_/TCS and M_L-A-E_/TCS) were determined against *S. aureus* ATCC 29213 and *E. coli* ATCC 25922 under pH 7.4 or 5.5 by a micro-dilution method [27, 28]. The experiments were performed in six replicates with the bacteria suspensions as the negative control.

##### Live/dead assay

The viability of the bacteria (ca. 10^7^ CFU/mL) treated with free TCS or TCS-loaded micelles was evaluated for different periods of time (1–12 h) at pH 5.5 or 7.4 using a LIVE/DEAD *Bac*Light Bacterial Viability Kit. After incubation, the bacteria were stained with a dye mixture of SYTO 9 dye and propidium iodide (1:1) at 25 °C for 30 min, and the fluorescent images were obtained using a Leica TCS SPE (Wetzlar, Germany).

#### Biofilm susceptibility

The effects of free TCS and TCS-loaded micelles on the mature biofilms of *E. coli* and *S. aureus* were discussed by CLSM. The biofilms were incubated with free TCS or TCS-loaded micelles (4–64 µg/mL, pH 7.4 or 5.5) for 24 h, and then stained with a LIVE/DEAD *BacLight* Bacterial Viability Kit. The residual biofilm images were obtained through a Zeiss LSM 880 microscopy (Zeiss, Germany). The Z-stack imaging was conducted using the areas near the center of the dishes at a 1-μm interval.

#### In vivo treatment of biofilms on catheters

The in vivo anti-biofilm efficiency of M_L-E-A_/TCS and M_L-A-E_/TCS was investigated on implant-related biofilm infection. Ten-mm segments of commercial catheters were incubated in *E. coli* suspensions at 37 °C for 96 h to establish mature biofilms, then washed twice with saline and subcutaneously implanted in the inner thigh of Sprague–Dawley rats under sterile environment. The rats were intravenously treated with 500 μL M_L-E-A_/TCS and M_L-A-E_/TCS or free TCS at a dose of 2 mg/kg once daily for 7 days. The body weight of the rats was recorded daily from the first day to the end of the treatment.

At the end of the experiment, the implanted catheters and surrounding tissues were retrieved after the rats were euthanatized. The biofilm eradication was evaluated through SEM observation (Zeiss EVO LS15, Oberkochen, Germany). The bacteria growth rate was determined by calculating the colony-forming units (CFUs) after the bacteria were dispersed under low energy sonication for 45 min and incubated at 37 °C for 12 h. In addition, the retrieved tissue and major organs (heart, liver, spleen, lung and kidney) were observed under an optical microscope after stained with H&E.

#### Statistical analyses

The data were presented as mean ± standard deviation (S.D). Student’s *t*-test was used to compare the difference between two groups. Statistical significance was defined as *P < 0.05, **P < 0.01, ***P < 0.001 and ****P < 0.0001.

## Results and discussion

### Incorporation of PAE in the triblock copolymers

**Fig. 2:**
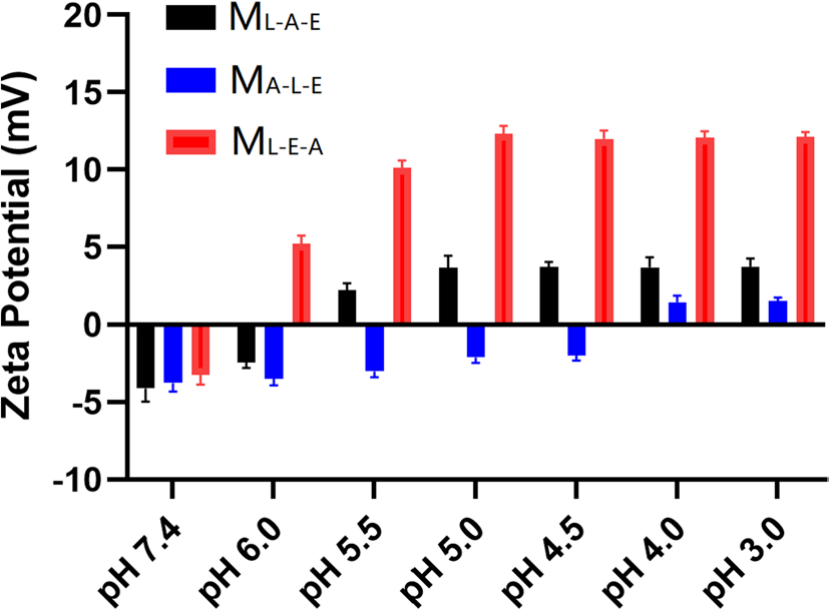
The pH_t_ of M_A-L-E_, M_L-A-E_ and M_L-E-A_

Due to the different pH between the vicinity of the biofilm (≈ 5.5) and healthy tissues (≈ 7.4), it is necessary for the pH-sensitive copolymers to have an appropriate triggering pH (pH_t_) around 5.5. The pH_t_ of M_A-L-E_, M_L-A-E_ and M_L-E-A_ was investigated by testing the pH-dependent zeta potential. As shown in Fig. 2, the pH_t_ of M_A-L-E_, M_L-A-E_ and M_L-E-A_ was around 4.0, 5.5 and 6.0. Previous studies indicated that the pK_b_ of PAE segments was around 6.5 [29, 30], which suggested that the distribution of PAE segments in the polymer chains would significantly affect its protonation capability. Among the tested copolymers, M_A-L-E_ showed the lowest pH_t_, which meant that M_A-L-E_ demanded more tertiary amine groups in the PAE segments protonated at much lower pH to switch the surface charge. This might be due to that the PAE segments distantly located from the micellar surface and separated with the PEG segments. Thus, PAE segments would be preferentially placed near the micellar surface and associated with PEG segments. This was significantly in that it would facilitate the PAE segments rising to the surface due to the relatively hydrophilicity under acidic conditions and association with the PEG. Additionally, the pH_t_ of M_L-A-E_ was a little lower than that of M_L-E-A_, which might be attributed to the additional hydrophobic force arising from the linkage with PLA segments. Due to the very acidic pH_t_ of M_A-L-E_, it is not suitable for targeting biofilms. Both M_L-A-E_ and M_L-E-A_ were found to display a desirable pH_t_ (~ 5.5), which could specifically respond to the biofilm environment pH, and selected for the next study.

### pH-dependent physicochemical properties of micelles

**Fig. 3:**
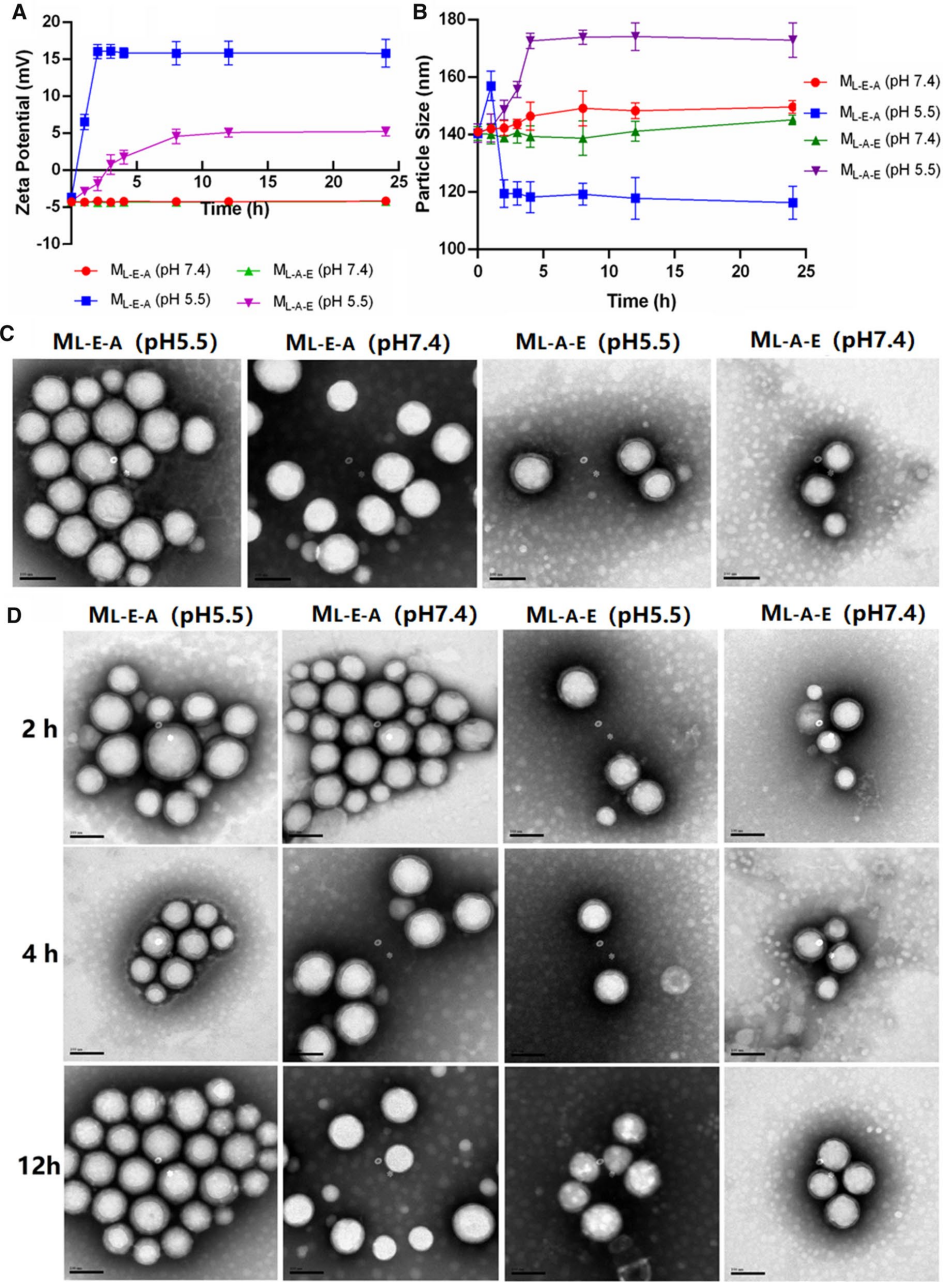
The variations in Zeta potentials (**A**) and particle sizes (**B**) of M_L-E-A_ and M_L-A-E_ vs time under pH 7.4 and 5.5, respectively. Error bars denote the standard deviations. TEM images of M_L-E-A_ and M_L-A-E_ obtained under pH 7.4 and 5.5 (**C**). The morphology changes of M_L-E-A_ and M_L-A-E_ under pH 7.4 and 5.5 with the time (**D**). Scale bars = 100 nm. N = 3 for all observations

The particle size and zeta potential of M_L-A-E_ and M_L-E-A_ was measured at different pH conditions to investigate the pH-dependent physicochemical properties. As shown in Fig. 3A, both M_L-A-E_ and M_L-E-A_ could keep negatively charged with minor changes at pH 7.4. The surface charge of M_L-E-A_ could quickly switch to a positive one (≈ + 16 mv) within 2 h at pH 5.5. In contrast, the zeta potential of M_L-A-E_ only switched from − 4.11 mV to + 4.74 mV at pH 5.5, and the transition took more than 4 h. We reasoned that the longer transform time and lower zeta potential transition of M_L-A-E_ was attributed to (1) compared with M_L-E-A_, the PAE segments in M_L-A-E_ needed to be more ionized in order to break the additional hydrophobic force arising from the close association with PLA segments, which needed more time. (2) After ionized fully, the outmost PEG shell would still cover the positive charged PAE^+^.

The size variation of M_L-A-E_ and M_L-E-A_ under different pH conditions was also measured with the incubation time extending (Fig. 3B). The particle size of both M_L-A-E_ and M_L-E-A_ showed no obvious change after incubation at pH 7.4 for 24 h, indicating the good stability at the physiological condition. Under pH 5.5, the particle size of M_L-E-A_ firstly increased within 1 h of incubation. The reason may be that PAE protonated and began to disassociate from the hydrophobic core. The micelles transformed from dense to loosen structure, which led to an increase of the particle size around 20 nm [31] Further extending the incubation time, the particle size started to decrease until 2 h, and then leveled off. This was probably because that the tertiary diamine moieties of PAE were fully ionized after 2 h of incubation, and the new cationic micelles (M_L-E-A_^+^) with PLA as the inner core and PEG/PAE^+^ as the mixed shell formed, and the corresponding size was around 120 nm. Obviously, the micelle size of M_L-E-A_ was a little bigger than that of M_L-E-A_^+^. This might be attributed to the more hydrophobic core content, the bigger particle size. In contrast, the particle size of M_L-A-E_ kept increasing with the incubation time extending to 4 h, which was also attributed to the protonation of PAE segments, leading to a loosen structure. Different from M_L-E-A_, further extending the incubation time, the particle size just stopped increasing without shrinking, and reached a plateau. This was probably because the electrostatic repulsion between the PAE^+^ segments induced the swelling of PAE residues. The closely associated PLA segments could not form tight inner core under this condition. TEM images demonstrated that M_L-A-E_ and M_L-E-A_ appeared round and smooth under both pH 7.4 and 5.5 (Fig. 3C). Further prolonging the incubation time, both M_L-A-E_ and M_L-E-A_ could retain the micellar integrity under acidic (Fig. 3D), which could enable the slow releasing behavior of the loaded drugs and tailor micelles-bacterium interactions.

### pH-dependent micelles-bacteria binding and penetration into biofilms

**Fig. 4:**
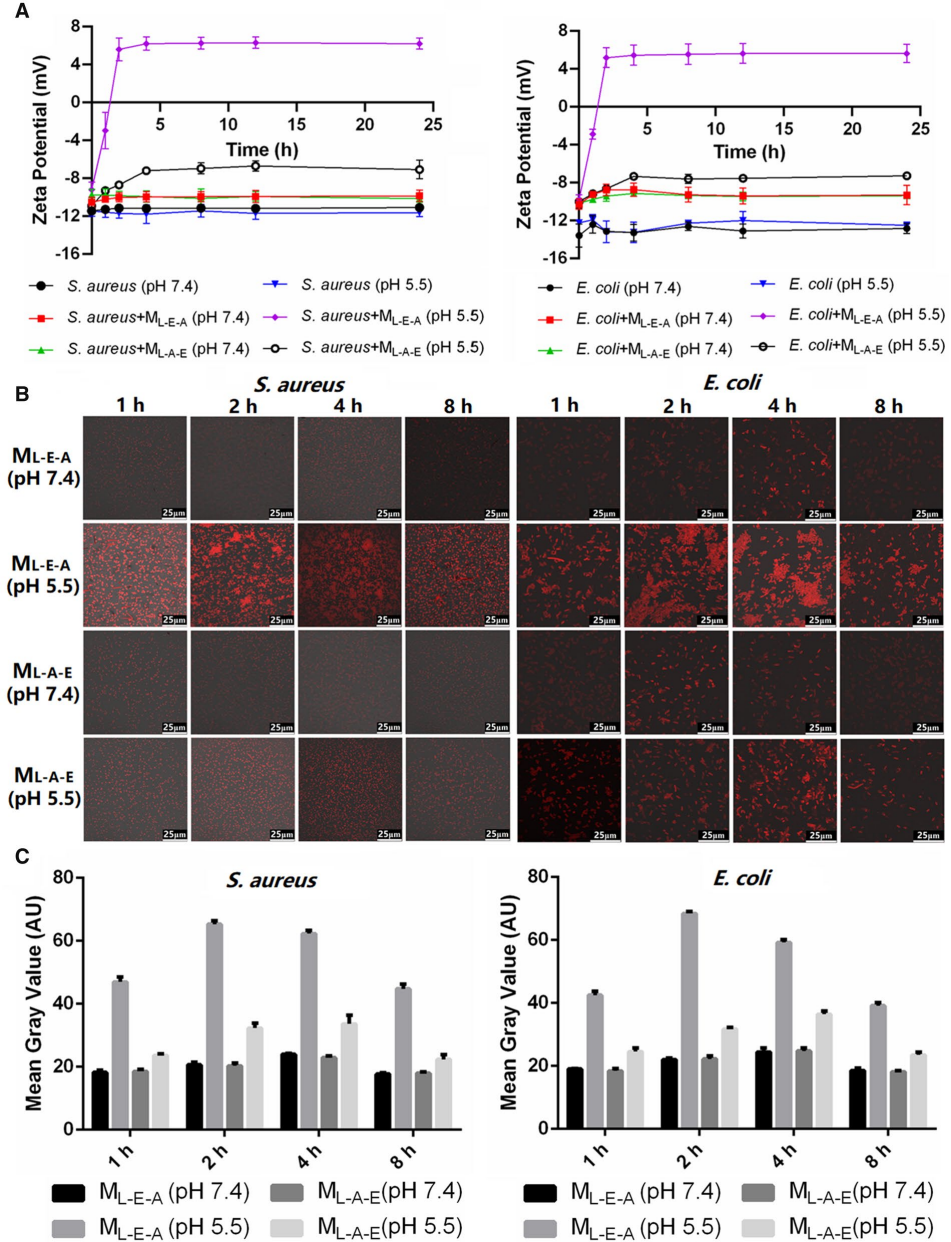

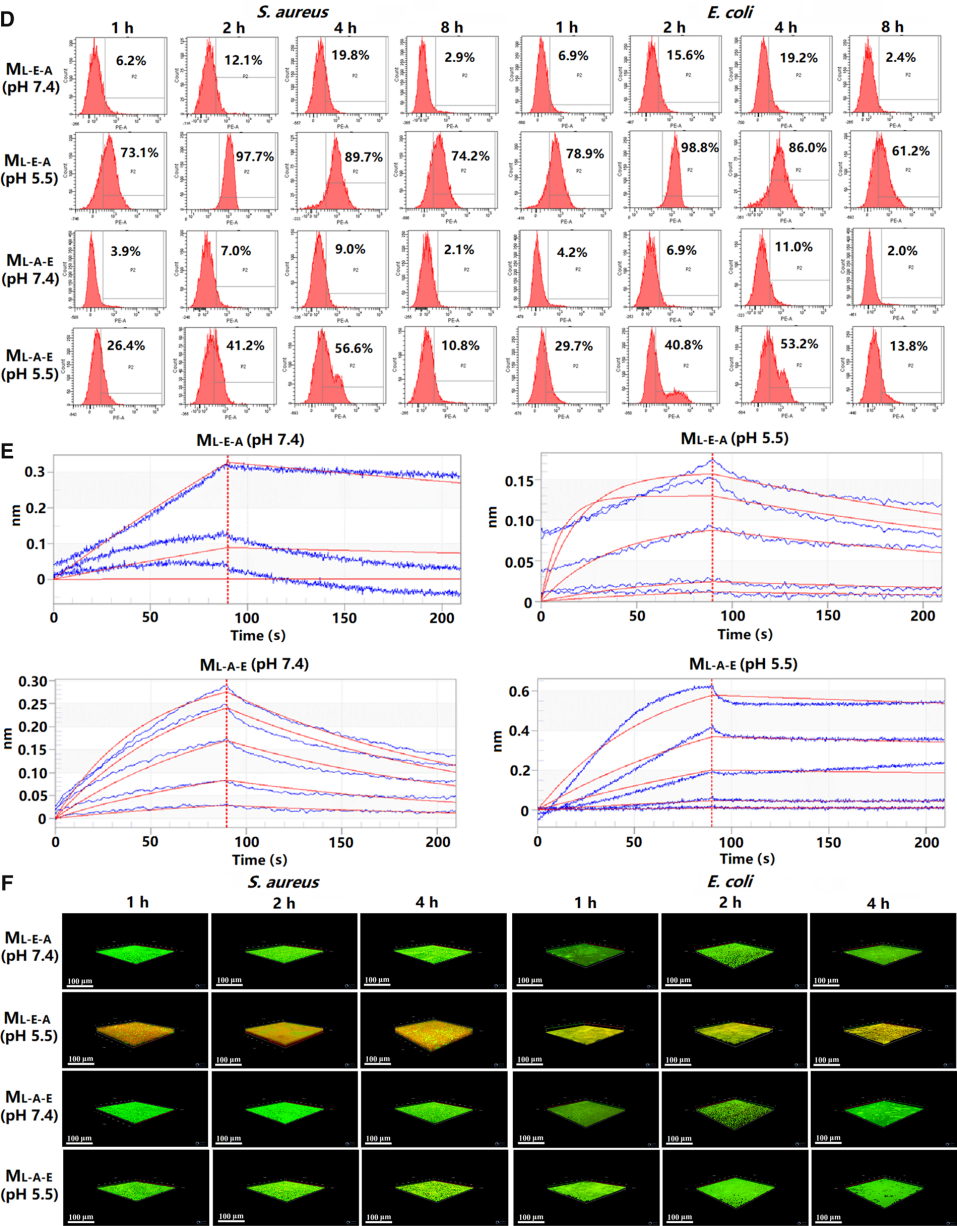
Zeta potential changes of *S. aureus* and *E. coli* after incubation with M_L-E-A_ and M_L-A-E_ under pH 7.4 or 5.5 for different periods of time, respectively (**A**). CLSM images of *S. aureus* and *E. coli* incubated with Nile Red loaded M_L-E-A_ and M_L-A-E_ for 1 h, 2 h, 4 h and 8 h, respectively (**B**). The mean gray value of CLSM images measured by Fiji ImageJ (**C**). Quantitative cellular uptake of M_L-E-A_/Nile red and M_L-A-E_/Nile red in *S. aureus* and *E. coli* evaluated by flow cytometry at pH 7.4 and 5.5 for 1 h, 2 h, 4 h and 8 h, respectively (**D**). BLI assay for the interaction between b-LPS and M_L-E-A_ (pH 7.4), M_L-A-E_ (pH 7.4), M_L-E-A_ (pH 5.5) and M_L-A-E_ (pH 5.5) (**E**). CLSM images of biofilms penetration of M_L-E-A_/Nile red and M_L-A-E_/Nile red at pH 5.5 and 7.4 after 1 h, 2 h and 4 h of incubation, respectively. Scale bars = 100 μm (**F**)

We wondered whether the different pH-dependent physicochemical properties of M_L-E-A_ and M_L-A-E_ could affect the binding affinity with bacteria, and then evaluated the micelles-bacterium interactions by a variety of experimental techniques. As shown in Fig. 4A, both *E. coli* and *S. aureus* remained pH-insensitive and negatively charged with the zeta potential around − 11 mv and − 12 mv, respectively. It could be observed that the binding affinity of M_L-E-A_ was markedly influenced by pH values. At pH 7.4, only a subtle increase of zeta potential was observed after incubation with M_L-E-A_ in the case of both bacteria, indicating the limited binding affinity. However, the zeta potential of the bacteria increased rapidly and even reversed to a positive one when the pH dropped to 5.5, suggesting a large and increase in binding. Oppositely, although M_L-A-E_ showed pH-dependent targeting towards bacteria, the interactions between them remained low at pH 5.5. This demonstrated that the pH-stimulated change in zeta potential and structure transition of micelles significantly affected the targeting ability to bacteria. For M_L-E-A_, the PAE moieties could be ionized and rise to the surface as the outermost layer at acidic condition, resulted in the positive charge density of the micelles surface dramatically increasing. The increased positive surface charge could facilitate M_L-E-A_^+^ targeting toward the negatively charged bacterial cell membrane. Although, the PAE segments in M_L-A-E_ could also protonated at pH 5.5, the weak positively charged surface and PEG shell would hamper the electrostatic interaction between cationic micelles and bacteria.

The pH-dependent binding behavior of M_L-A-E_ and M_L-E-A_ to bacteria was further evaluated by CLSM. CLSM visually confirmed the pH-sensitive nature of M_L-A-E_ and M_L-E-A_ binding to bacteria (Fig. 4B). It could be seen that the red fluorescence of both micelles concurrently increased with the decrease of pH. Strong fluorescence could be seen at the pH 5.5 groups but not pH 7.4 groups. Moreover, the pH-dependent increase in binding affinity of M_L-E-A_ was more pronounced as compared to that of M_L-A-E_. After 2 h of incubation at pH 5.5, the M_L-E-A_/Nile red showed the strongest red fluorescence with the mean gray value around 65 AU, and the red fluorescence could last more than 8 h (Fig. 4C). Additionally, at the acidic condition, large aggregates of bacteria were observed after treated with M_L-E-A_. This might be attributed to the bridge effect of positively charged M_L-E-A_^+^ trigging the negatively charged bacteria agglutination. The influence of pH on the binding ability of micelles with bacteria was further quantitatively investigated by flow cytometry. As shown in Fig. 4D, in line with the CLSM observation, a large amount of M_L-E-A_ could be taken up by *E. coli* and *S. aure*us at pH 5.5, and peaked at 2 h.

Based on these results, we believed that the protonated PAE^+^ segments played a critical role in binding to bacteria by electrostatic interacting with the negatively charged component of bacteria, e.g*.* lipopolysaccharide (LPS) or peptidoglycan (PGN). The binding affinity of M_L-A-E_ and M_L-E-A_ with LPS, one of the main structural components of the Gram-negative bacteria, was monitored and quantified using BLI at pH 5.5 and 7.4, respectively. According to the observed kinetics (Fig. 4E), the interactions between M_L-E-A_ and LPS was significantly affected by the environment pH. Under physiological pH, the M_L-E-A_ showed less affinity to LPS with an affinity constant (K_D_) of 1.57 × 10^–5^ M, which was similar as that of M_L-A-E_ (K_D_, 4.82 × 10^–5^) (Table 1). This stated that the negatively charged surface and PEG-shell would impede the micelles-LPS interactions due to the electrostatic repulsion and steric hindrance. When the pH lowered to 5.5, the interactions between M_L-E-A_ and LPS significantly increased, and the K_D_ decreased to 6.03 × 10^–7^ M. However, there was no significant difference in M_L-A-E_ over the pH trajectory. Thus, the enhanced binding affinity of M_L-E-A_ under acidic condition could be due to the increased interactions with LPS. It was possible that M_L-E-A_ may act in a similar way to *S. aureus* by interacting with the negatively-charged component of cell wall (e.g. peptidoglycan).

**Table 1:** Binding parameters of b-LPS with M_L-E-A_ and M_L-A-E_ under different pHs measured by BLI

Analyte	k_on_ (1/Ms)	k_dis_ (1/s)	K_D_ (M)
M_L-E-A_ (pH 7.4)	4.61E+02	7.25E−03	1.57E−05
M_L-A-E_ (pH 7.4)	3.35E+01	1.61E−03	4.82E−05
M_L-E-A_ (pH 5.5)	1.03E+03	6.23E−04	6.03E−07
M_L-A-E_ (pH 5.5)	3.07E+03	3.21E−03	1.05E−06

The stealth penetration and accumulation capability of M_L-A-E_ and M_L-E-A_ in biofilms were further evaluated using *S. aureus* and *E. coli* as the model organisms. The biofilms were incubated with the Nile red loaded micelles for 1 h, 2 h and 4 h, and then stained by STYO 9. As depicted in Fig. 4F, demonstrable penetration and accumulation of red-fluorescent M_L-E-A_ and M_L-A-E_ at pH 7.4 was completely absent. At pH 5.5, M_L-E-A_ and M_L-A-E_ showed a completely different pattern of penetration of accumulation in biofilms. M_L-E-A_/Nile red penetrated well into the biofilms, while rapid saturation of fluorescence and deep accumulation even to the biofilm bottom occurred within 1 h for both bacterial biofilms. Whereas M_L-A-E_ did not show obvious sign of increased penetration and accumulation in biofilms at pH 5.5. Evidently, the targeted interaction of M_L-E-A_ with bacteria allowed the penetration and accumulation into biofilms at a low pH condition by avoiding from being washed out.

### Characterization of TCS-loaded micelles

**Fig. 5:**
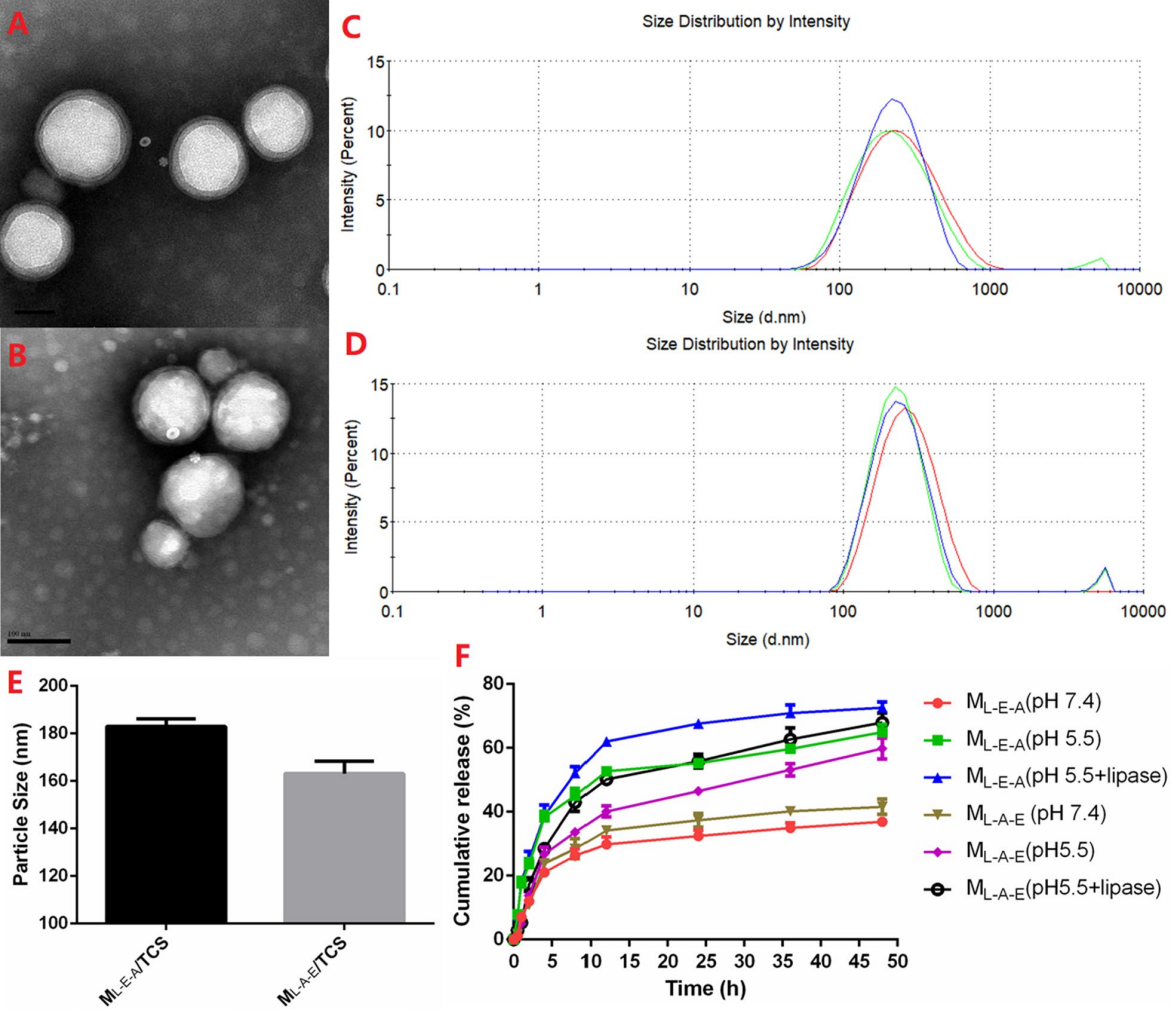
Morphology of M_L-E-A_/TCS (**A**) and M_L-A-E_/TCS (**B**) obtained by TEM (n = 3). Scale bars = 100 nm. The particle size and distribution of M_L-E-A_/TCS (**C**) and M_L-A-E_/TCS (**D**) obtained by DLS. The particle sizes of ML-E-A/TCS and ML-A-E/TCS (**E**). The cumulative release of TCS from M_L-E-A_/TCS and M_L-A-E_/TCS at pH 7.4, pH 5.5 or pH 5.5 in presence of lipase, respectively (**F**)

A spherical and homogeneous morphology of both M_L-E-A_/TCS and M_L-A-E_/TCS was observed by TEM (Fig. 5A, B), and the particle size was consistent with that obtained by DLS (Fig. 5C, D). Moreover, the characteristics of the TCS-loaded M_L-E-A_ and M_L-A-E_ at pH 7.4 were summarized in Table 2. Blank M_L-A-E_ and M_L-E-A_ had a similar diameter of around 140 nm (Fig. 3C), while the diameters increased to 180 nm and 160 nm after TCS being loaded (Fig. 5E). Both M_L-E-A_/TCS and M_L-A-E_/TCS were slightly negatively charged at pH 7.4. The nearly net surface charge would enable the micelles escaping from the opsonin recognization, and passively targeting the infection sites [32].

**Table 2:** The physicochemical characteristics of different micellar formulations (n = 3)

Formulations	Particle size (nm)	Zeta potential (mv)	PDI	DL%	EE%
M_L-E-A_/TCS	182.97 ± 3.12	− 1.95 ± 0.398	0.242 ± 0.011	7.92 ± 0.21	77.45 ± 5.67
M_L-A-E_/TCS	163.13 ± 5.23	− 2.64 ± 0.149	0.210 ± 0.042	8.05 ± 0.24	79.12 ± 4.56

The in vitro release performances of M_L-E-A_/TCS and M_L-A-E_/TCS were evaluated under different conditions. It could be observed that under physiological condition, both micelles exhibited a sustained release pattern, only 40% of TCS released after 48 h. When the pH was lower, the drug release was accelerated, and the cumulative release was over 60% for both M_L-E-A_/TCS and M_L-A-E_/TCS. This could be due to the protonation of amino groups in PAE moieties at lower pH conditions leading to the micelle structure loose. Bacterial enzymes such as lipase can promote the hydrolysis of the PLA core, which may accelerate the release of the loaded drugs. In the presence of lipase, the release of TCS from both M_L-E-A_/TCS and M_L-A-E_/TCS increased, with an overall release rate around 70% after 48 h. Our results indicated that the loosen hydrophobic core of micelles under pH 5.5 was amenable to enzymatic degradation, leading to a higher drug release rate. Thus, the stronger lipase liability in the drug release might enhance the biofilm destruction of TCS-loaded micelles.

### Effect of TCS and TCS-loaded micelles on the growth of planktonic and biofilm bacteria

We were also interested in whether the enhanced pH-dependent binding affinity of M_L-E-A_ had an impact on its in vitro antibacterial efficacy and biofilm eradication and how this compared to M_L-A-E_ and free TCS. The MICs of M_L-E-A_/TCS, M_L-A-E_/TCS and free TCS for planktonic *E. coli* and *S. aureus* at pH 7.4 and 5.5 were showed in Table 3. As expected, both bacteria appeared more susceptible to free TCS than to encapsulated TCS at pH 7.4, and free TCS was the most potent agent against planktonic bacteria. At pH 5.5, the antibacterial activity of M_L-E-A_/TCS was significantly improved, achieving a similar antibacterial effect compared with that of free TCS at the same concentration. Oppositely, the changing of pH did not influence the antibacterial activity of M_L-A-E_/TCS dramatically. The difference between MIC (pH 5.5) and MIC (pH 7.4) was very small and only involved one dilution steps.

**Table 3:** Antibacterial activities of the tested formulations

Formulations	MIC (µg/mL)			
	*S. aureus*		*E. coli*	
	pH 7.4	pH 5.5	pH 7.4	pH 5.5
Triclosan	1.0	2.0	4.0	4.0
M_L-E-A_/TCS	8.0	1.0	32.0	4.0
M_L-A-E_/TCS	8.0	4.0	32.0	16.0

**Fig. 6:**
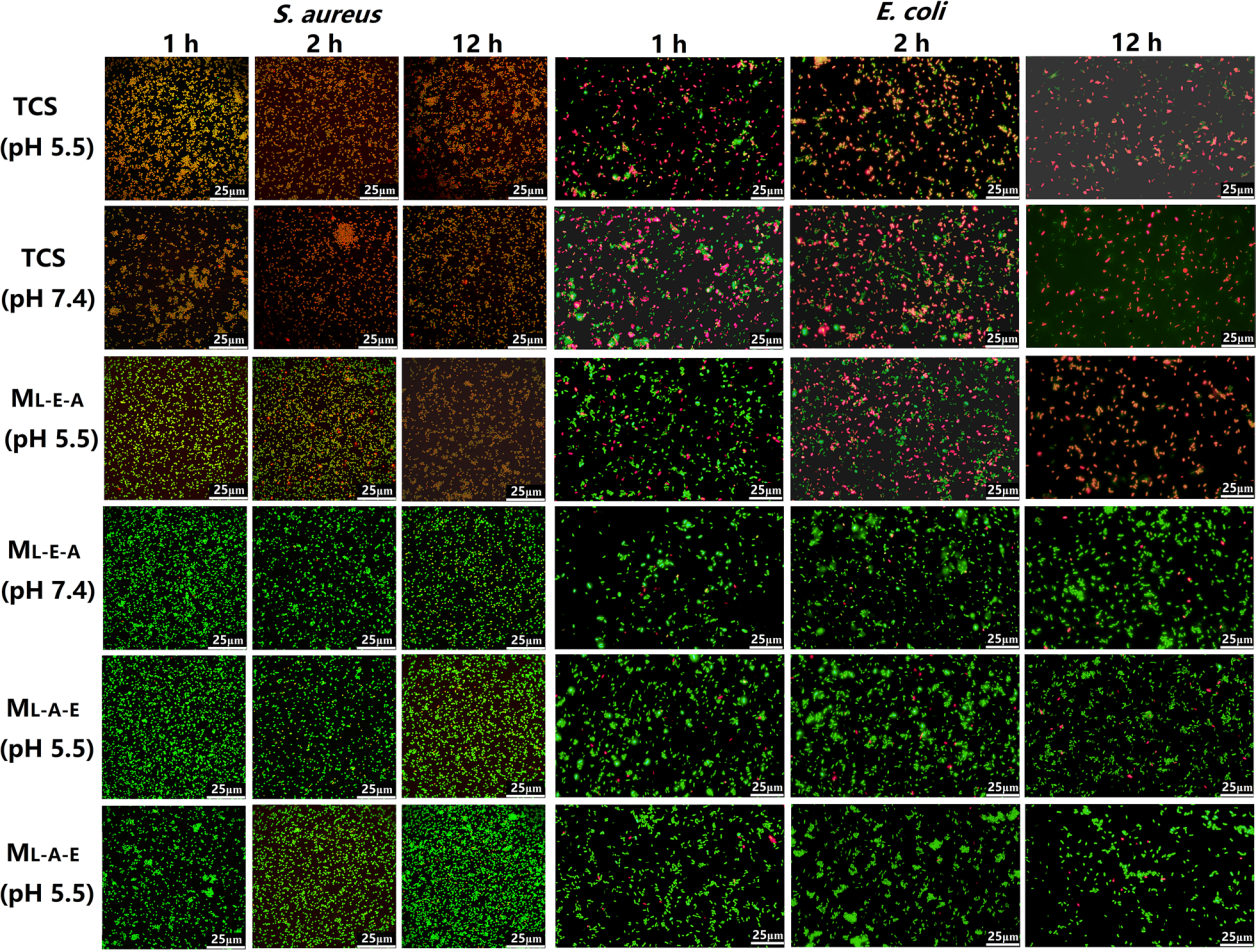
Fluorescent live/dead images of *E. coli* and *S. aureus* after incubation with free TCS, M_L-E-A_/TCS and M_L-A-E_/TCS under pH 7.4 and 5.5 at 37 °C for different periods of times (1–12 h). Scale bars = 25 μm

The pH-dependent bacterial viability after the treatment with free TCS, M_L-E-A_/TCS and M_L-A-E_/TCS was further measured using PI (red) and SYTO 9 (green). All the cells could be stained with green-fluorescent STYO 9. Only the dead ones with compromised membrane were stained red, and yielded yellow-fluorescent images. From the Fig. 6, most of the cells (both *E. coli* and *S. aureus*) treated with free TCS were fluorescing yellow at an exposure time of 1 h, suggesting that most bacterial cells were dead within 1 h. M_L-E-A_/TCS showed comparable antibacterial effects after an exposure time of 2 h at pH 5.5, but was ineffective at pH 7.4. This demonstrated that the surface charge changes of M_L-E-A_ from negative to positive at the acidic environment could effectively enhance the bacterial capacity. Obviously, the bactericidal efficiency of M_L-E-A_/TCS was time-needed compared with the effects of free TCS, since the protonation of PAE segments, enzymatic degradation of PLA core and releasing of TCS required time. Corresponding with MIC results, pH value did not play an important role in the antibacterial efficiency of M_L-A-E_/TCS.

**Fig. 7:**
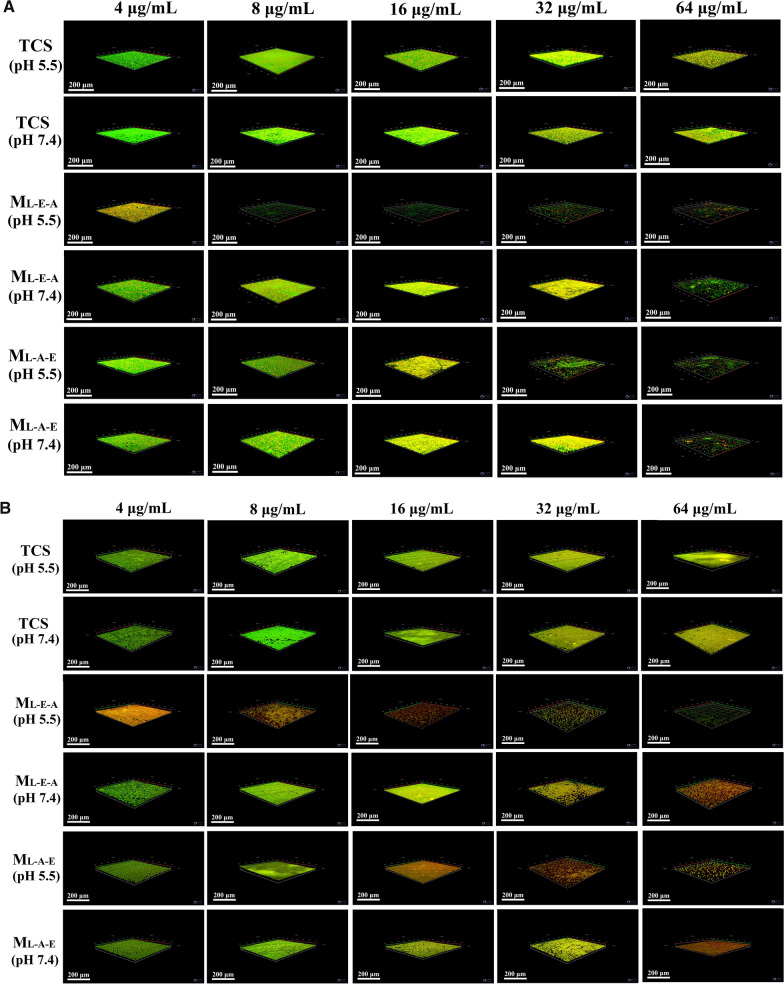
CLSM analysis of *S. aureus* (**A**) and *E. coli* (**B**) biofilm eradication. The *S. aureus* and *E. coli* biofilms were treated with free TCS, M_L-E-A_/TCS and M_L-A-E_/TCS at different concentrations overnight

Next, the killing efficacy of free TCS and TCS-loaded micelles was compared against bacteria in their biofilm mode of growth through CLSM observation, and the biofilm residues were also stained with live/dead staining. As illustrated in Fig. 7, free TCS showed limited effect against mature biofilms even at the highest concentration of 64 μg/mL at both pHs. Although the yellow-fluorescence was concentration-dependent, which indicated the decrease in the cell viability, the morphology of the biofilms had no change. Encapsulation TCS into micelles could promote its anti-biofilm activity, and a completely biofilm eradication could be observed at a concentration of 64 μg/mL for both M_L-A-E_/TCS and M_L-A-E_/TCS at pH 7.4. Under acidic condition, the biofilm eradication ability of both micelles was further improved, especially for the effects of M_L-E-A_/TCS. A dramatic decrease in biofilm thickness and bacterial viability was obtained at a lower concentration of 8 μg/mL against both *E. coli* and *S. aureus*. Infection control of bacteria in biofilms has been plagued for many years. Free TCS has shown to be effective against planktonic bacteria, but not those in the biofilm mode. Although the encapsulated TCS showed reduced antibacterial activity against planktonic bacteria, the advantages with respect to biofilm eradication became evident especially under pH 5.5. M_L-E-A_/TCS could efficiently penetrate and accumulate in the biofilm to reach and kill the bacteria inside of the biofilm at acidic conditions. This could be due to that, upon the initial penetration, the pH-sensitive PAE segments of M_L-E-A_ efficiently switched to be positively charged and rose to the surface, which could effectively engage the interaction with the negatively charged bacteria in the biofilms [25, 33–35]. Subsequently, by close contact with the bacteria, M_L-E-A_ was able to be degraded by lipase, leading to a quick release of the loaded antimicrobial drug [36]. As an overall result, the antibacterial effect of micelles could be partially enhanced by promoting micelles-bacterium interactions at acidic pH, further demonstrating the delivery system based on M_L-E-A_ has great potential in the treatment of infections with localized acidity.

### In vivo anti-biofilm effects on catheters

**Fig. 8:**
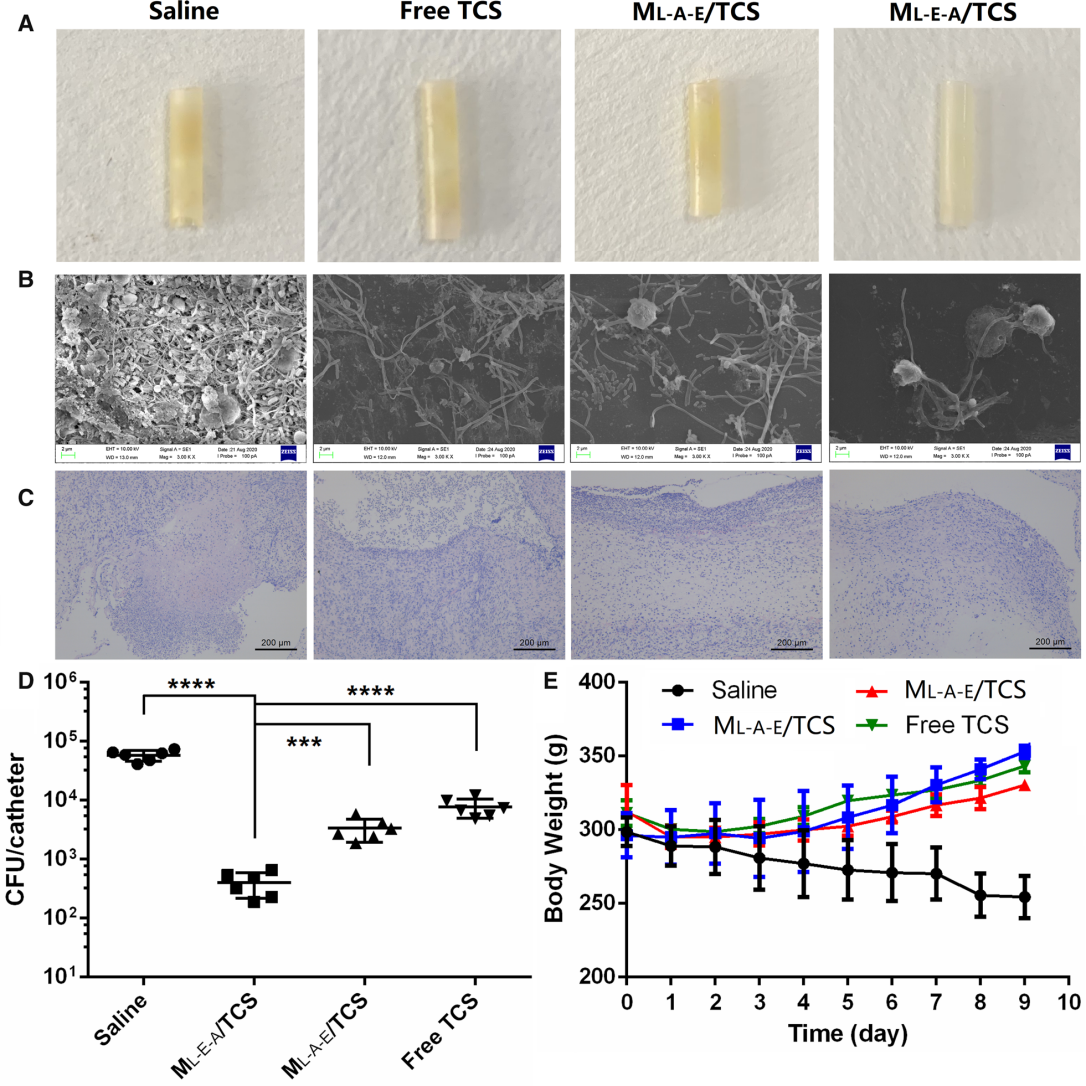
In vivo treatment of biofilms formed on subcutaneously implanted catheters. Images of the catheters (**A**), SEM images of residual biofilms (**B**), H&E images of the tissues around the implanted catheters (**C**) the residual bacterial in the biofilms (**D**), and the body weight changes of SD rats (**E**) after treatment for 5 days with saline, free TCS, M_L-A-E_/TCS and M_L-E-A_/TCS, respectively

Implant-related infection has been associated with bacterial colonization and biofilm formation during artificial implants [37]. In this section, the in vivo eradication effects of free TCS and TCS-loaded micelles on the subcutaneously implanted catheters covered with *E. coli* biofilms were investigated in vivo. The catheters showed no signs of contamination and color changes after 5 days of M_L-E-A_/TCS treatment (Fig. 8A). SEM observation also indicated that there was no obvious biofilm on the surface of catheters. The established biofilm on catheters could be significantly disrupted and the embedded aggregated bacteria could be eradicated by M_L-E-A_/TCS treatment (Fig. 8B). By contrast, the catheter surface was greatly contaminated with bacteria, and biofilms were apparently covered after the saline treatment. Although the treatment by M_L-A-E_/TCS and free TCS could alleviate the contamination levels on the catheters, the biofilms could not be eradicated completely. Moreover, a remarkable reduction of tissue lesions (Fig. 8C), decreased CFU (Fig. 8D) and recovered body weight (Fig. 8E) further confirmed the inflammatory disappearance after treatment with M_L-E-A_/TCS in comparation with free TCS and M_L-A-E_/TCS. The symptoms of serious infection, including local redness and swelling, weight loss, anorexia and fever, could not be observed in M_L-E-A_/TCS group.

**Fig. 9:**
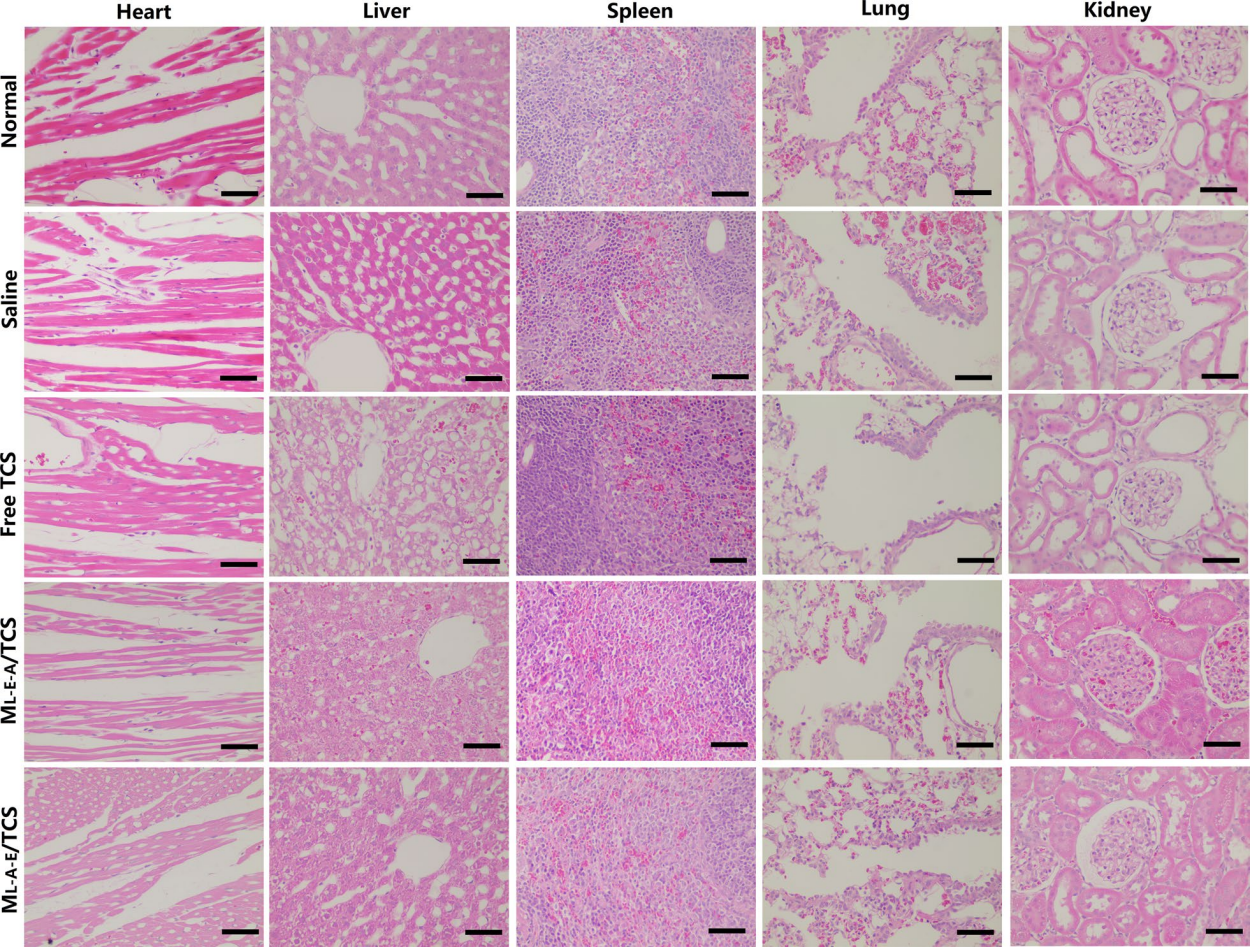
H&E staining images of heart, liver, spleen, lung and kidney of healthy rats and implant-related biofilm infection rats after treatment with saline, free TCS, M_L-E-A_/TCS and M_L-A-E_/TCS for 7 days. Scale bars = 50 μm

No significant in vitro cytotoxicity (Additional file 1: Fig. S8A) and hemolytic behavior (Additional file 1: Fig. S8B) was observed for both M_L-E-A_/TCS and M_L-A-E_/TCS. Moreover, the H&E staining images of heart, liver, spleen, lung and kidney (Fig. 9) suggested that no obvious lesions or damage were observed in those tissues in both TCS-loaded micelles treated group, suggesting good biocompatibility. Conversely, free TCS caused fatty degeneration of hepatocytes, which was consistent with previous study. [38] Encapsulation of TCS in micelles could significantly lower its hepatotoxicity. These finding indicated that treatment of implant-related infection with M_L-E-A_/TCS could suppress bacterial growth, eradicate biofilms, lower the degree of infection with negligible side effects.

## Conclusions

Due to the increase of persistent bacterial infection, new enhanced strategies are urgently needed. In this study, we applied three types of pH-sensitive copolymers, PLA-PAE-mPEG, PAE-PLA-mPEG and PLA-PEG-PAE, which could self-assemble into three pH-sensitive surface charge-adaptive micelles (M_L-A-E_, M_A-L-E_ and M_L-E-A_) against biofilm-related infection, and focused on their structure–function relationship. Compared with M_L-A-E_ and M_A-L-E_, M_L-E-A_ showed superior capabilities in switching surface charge, binding to bacteria, penetrating biofilms, killing bacteria in deeper layers of a biofilm in vitro, and more efficiency in curing biofilm-related infection in vivo. In summary, the study indicated that rational design the architecture of pH-sensitive copolymers to maximize the charge-switching capability need to be considered when they were constructed for biofilm treatment. Due to the stealth performance and potential surface adaptive ability of the M_L-E-A_, the resistance of biofilms could be bypassed, thereby providing a potentially effective strategy against EPS-producing bacteria in its biofilm form.

## Supplementary Information

**Additional file 1**: **Fig. S1** Detailed synthetic route of PLA_5K_-PEG_5K_-PAE_5K_. **Fig. S2** Detailed synthetic route of PLA_5K_-PAE_5K_-mPEG_5K_. **Fig. S3** Detailed synthetic route of PAE_5K_-PLA_5K_-mPEG_5K_. **Fig. S4 **^1^H NMR spectrum of PLA_5K_-PEG_5K_-PAE_5K_. **Fig. S5 **^1^H NMR spectrum of PLA_5K_-PAE_5K_-mPEG_5K_. **Fig. S6 **^1^H NMR spectrum of PAE_5K_-PLA_5K_-mPEG_5K_. **Fig. S7 **The typical GPC spectrum of PAE_5K_-PLA_5K_-mPEG_5K_, PLA_5K_-PEG_5K_-PAE_5K_ and PLA_5K_-PAE_5K_-mPEG_5K_. **Fig. S8 **The *in vitro* cytotoxicity (A) and hemolysis behavior (B) of M_L-E-A_/TCS and M_L-A-E_/TCS as a function of TCS concentration (mean±SD, n=6). **Table S1** The characteristic of synthesized copolymers.

## Data Availability

All data generated or analysed during this study are included in this published article.
