# Supplementary material for: Elaboration on the architecture of pH-sensitive surface charge-adaptive micelles with enhanced penetration and bactericidal activity in biofilms
Source: J Nanobiotechnology. 2021 Aug 6;19:232. doi: 10.1186/s12951-021-00980-8 (PMC8344171; doi:10.1186/s12951-021-00980-8)
Supplement: Supplementary file 1 — Additional file 1: Fig. S1 Detailed synthetic route of PLA5K-PEG5K-PAE5K. Fig. S2 Detailed synthetic route of PLA5K-PAE5K-mPEG5K. Fig. S3 Detailed synthetic route of PAE5K-PLA5K-mPEG5K. Fig. S4 1H NMR spectrum of PLA5K-PEG5K-PAE5K. Fig. S5 1H NMR spectrum of PLA5K-PAE5K-mPEG5K. Fig. S6 1H NMR spectrum of PAE5K-PLA5K-mPEG5K. Fig. S7 The typical GPC spectrum of PAE5K-PLA5K-mPEG5K, PLA5K-PEG5K-PAE5K and PLA5K-PAE5K-mPEG5K. Fig. S8 The in vitro cytotoxicity (A) and hemolysis behavior (B) of ML-E-A/TCS and ML-A-E/TCS as a function of TCS concentration (mean±SD, n=6). Table S1 The characteristic of synthesized copolymers. [file 12951_2021_980_MOESM1_ESM.docx]

***Additional file 1***

**Elaboration on the Architecture of pH-sensitive Surface Charge-Adaptive Micelles with Enhanced Penetration and Bactericidal Activity in Biofilms**

Rong Guo^1^, Keke Li^1^, Baocheng Tian, Changrong Wang, Xiangjun Chen, Xinyu Jiang, Huayu He, Wei Hong^*^

*School of Pharmacy, Shandong New Drug Loading & Release Technology and Preparation Engineering Laboratory, Binzhou Medical University,* *346 Guanhai Road, Yantai, 264003, P. R. China.*

^*^*Corresponding author. Tel./ Fax.: +86-0535-6913718*

*E-mail address: hongwei_sy@* *bzmc.edu.cn*

**1. Experimental Section**

**1.1 Synthesis of PLA_5K_-PEG_5K_-PAE_5K_**

Firstly, PLA_5K_-COOH was synthesized by esterification reaction. Briefly, PLA (2.0 g), succinic anhydride (1.5 eq.) and SMAP (0.1 eq.) were dissolved in 20 mL anhydrous chloroform. The reaction was carried out at 50 °C for 24 h. The solvent was removed by rotary evaporation. The crude product was purified by precipitating in ice diethyl ether for three times. Finally, the precipitate was centrifuged and dried in vacuum to obtain PLA_5K_-COOH.

PLA_5K_-PEG_5K_-NH_2_ was obtained by amidation. PLA_5K_-COOH (1.0 g), EDC (3.0 eq.) and DMAP (0.1 eq.) were dissolved into 10 mL DMF, and stirred for 1 h. Then, the mixture solution was drop added into NH_2_-PEG_5K_-NH_2_ (3 eq.) dissolved in 5 mL DMF. The reaction was administrated at 40 °C for 24 h. The solution was then dialyzed (molecular weight 8000) against Milli-Q water for 48 h to remove unreacted NH_2_-PEG_5K_-NH_2_. The final product was then freeze-dried to obtain PLA_5K_-PEG_5K_-NH_2_.

1.0 g of PLA_5K_-PEG_5K_-NH_2_ was dissolved in 10 mL of dichloromethane, and then triethylamine (1.5 eq.) was added. Under the ice bath, 5 mL of acryloyl chloride (1.2 eq.) dichloromethane solution was dropwise added while stirring, and the mixture was stirred for 24 h. Then, the mixture was suction filtration and washed three times with distilled water, and the organic phase was collected to dry with anhydrous sodium sulfate. After vacuum concentration, the product was precipitated in a large amount of ice ether. The precipitate was centrifuged and dried in vacuum to obtain acrylated PLA_5K_-PEG_5K_-NH_2_.

1.0 g of acrylated PLA_5K_-PEG_5K_-NH_2_ was dissolved in 10 mL of chloroform, 1,4-butanediol diacrylate (10 eq.) and 1,3-bis (4-piperidinyl) propane (12 eq.) were added to dissolve completely. The mixture was reacted at 55 °C for 72 h. After vacuum concentration, the product was precipitated in ice ether. The precipitate was suction filtration and dried in vacuum to obtain PLA_5K_-PEG_5K_-PAE_5K_.

The detailed synthetic route was shown in Fig. S1.


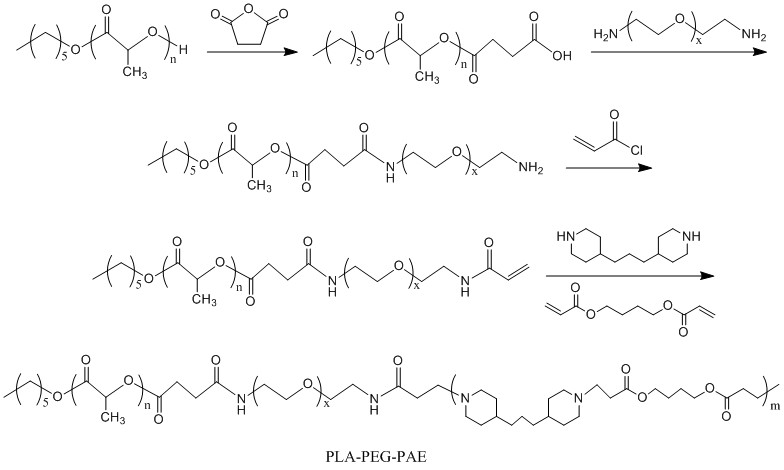


**Fig. S1** Detailed synthetic route of PLA_5K_-PEG_5K_-PAE_5K_

**1.2 Synthesis of PLA_5K_-PAE_5K_-mPEG_5K_**

PLA_5K_ (2.0 g) and trimethylamine (1.5 eq.) were dissolved in dichloromethane (20 mL) and placed in ice bath. The solution of dichloromethane 5 mL contained acryloyl chloride (1.2 eq) was drop added. After reacting for 24 h, suction filtration and washing were carried out to obtain crude product. The acrylated PLA_5K_ was got through precipitate in ice diethyl ether.

Acrylated PLA_5K_ (1.0 g), 1,4-butanediol diacrylate (10 eq.) and 1,3-di (4-piperidinyl) propane (12 eq.) were dissolved in 10 mL of chloroform. The reaction was maintained for 72 h at 55 °C. After polymerization, the crude product PLA_5K_-PAE_5K_ was precipitated into an excess amount of ice ether. The product was centrifuged and dried in vacuum to obtain pure PLA_5K_-PAE_5K_.

PLA_5K_-PAE_5K_ (1.0 g) and N-acryloyloxysuccinimide (10 eq.) were dissolved in 10 mL DMF. The mixture was stirred at 55 °C for 12 h. The product was obtained by precipitating into excess ice ether. After centrifugation and vacuum drying, PLA_5K_-PAE_5K_-NHS was obtained.

PLA_5K_-PAE_5K_-NHS (1.0 g), mPEG_5K_-NH_2_ (2.0 eq.) and triethylamine (5.0 eq.) were dissolved in 10 mL DMF. Then, the reaction was administrated for 24 h at room temperature. Finally, the solution was transferred to dialysis bag (molecular weight 8000) and dialyzed with pure water for 48 h. The dialysate was collected and freeze-dried to obtain the product of PLA_5K_-PAE_5K_-mPEG_5K_.

The detailed synthetic route was shown in Fig. S2.


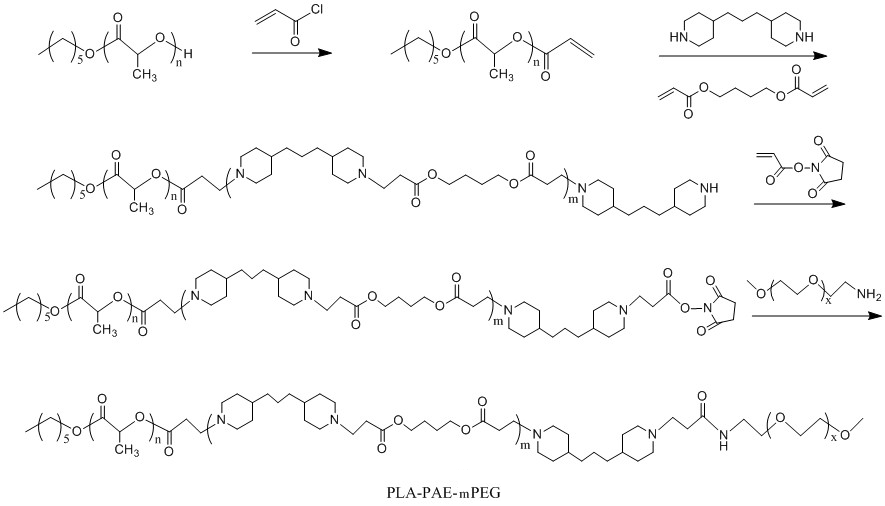


**Fig. S2** Detailed synthetic route of PLA_5K_-PAE_5K_-mPEG_5K_

**1.3 Synthesis of PAE_5K_-PLA_5K_-mPEG_5K_**

mPEG_5K_-OH (1.0 g), lactide and catalytic (1.0 g) and stannous octanoate were dissolved in 15 mL of anhydrous toluene and reacted at 130 °C for 24 h under the protection of nitrogen. The product was obtained by precipitating into excess ice ether. After centrifugation and vacuum drying, OH-PLA_5K_-mPEG_5K_ was obtained.

1.0 g of OH-PLA_5K_-mPEG_5K_ was dissolved in 10 mL of anhydrous dichloromethane, and then acryloyl chloride (5.0 eq.) and triethylamine (5.0 eq.) were added. The mixture was stirred at room temperature for 24 h. Then, the solution was concentrated in a rotary evaporator, and washed three times with distilled water. The organic phase was collected to dry with anhydrous sodium sulfate. The product was precipitated in ice ether. After centrifugation and vacuum drying, propylene-PLA_5K_-mPEG_5K_ was obtained.

Propylene-PLA_5K_-mPEG_5K_ (0.5 g) was dissolved in chloroform (10 mL), and then 1,3-bis (4-piperidine) propane (11 eq.), 1,3-bis (4-piperidine) propane and 4-bis (acryloyloxy) butane (10 eq.) were added. The reaction was maintained at 55 °C for 72 h, and the solution was poured into an excess of ethyl ether. The final product of PAE_5K_-PLA_5K_-mPEG_5K_ was obtained by filtration and vacuum drying for 2 days at room temperature.

The detailed synthetic route was shown in Fig. S3.

**
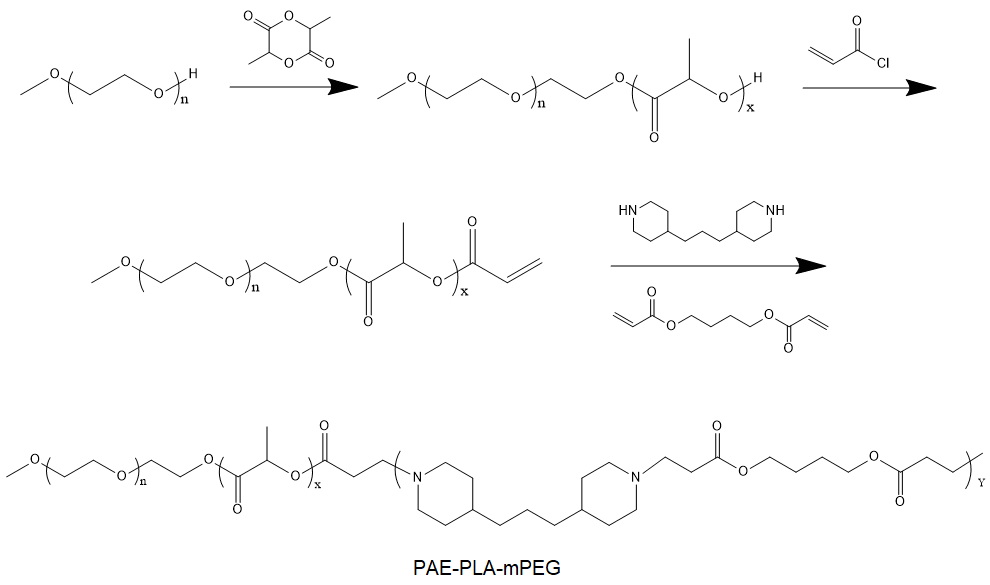
**

**Fig. S3** Detailed synthetic route of PAE_5K_-PLA_5K_-mPEG_5K_

**1.4 Characterization of copolymers**

The chemical structure of the PLA_5K_-PAE_5K_-mPEG_5K_, PLA_5K_-PEG_5K_-PAE_5K_ and PAE_5K_-PLA_5K_-mPEG_5K_ copolymers were characterized by a Bruker DRX-600 NMR instrument at 600 MHz. The molecular weight and distribution of PLA_5K_-PAE_5K_-mPEG_5K_, PLA_5K_-PEG_5K_-PAE_5K_ and PAE_5K_-PLA_5K_-mPEG_5K_ were measured by a gel permeation chromatography (GPC) system. Polyethylene glycol (PEG) was used as standard polymer, while chloroform was used as eluent with a flow rate of 1 mL/min at 40 °C. The number average molecular weight of PLA block and PAE block was calculated using equation (1) and (2), respectively:

$M_{n}\left( \mathrm{PLA} \right)=A_{\mathrm{PLA}}\times5000\times72\times4/A_{\mathrm{PEG}}\times44\times1$ (1)

Where A_PLA_ and A_PEG_ is the peak area of PLA block and PEG block according to the ^1^H NMR results, respectively.

$M_{n}\left( \mathrm{PAE} \right)=M_{n}\left( \mathrm{copolymer} \right)-M_{n}\left( \mathrm{PLA} \right)-M_{n}(PEG)$ (2)

Where M_n_ (copolymer) is the number average molecular weight of PLA-PEG-PAE, PLA-PAE-mPEG and PAE-PLA-mPEG determined by GPC, respectively. M_n_ (PLA) is the number average molecular weight of PLA block calculated from ^1^H NMR. The number average molecular weight of M_n_ (PEG) is 5000.

**1.5 *In vitro* cytotoxicity and hemolysis test**

Vero cells were seeded in 96-well TCPs overnight, followed by incubation with different concentrations of TCS-loaded micelles (with a final TCS concentration of 20 ~ 160 μg/mL) for 24 h. The cell viability of Vero cells after treated with M_L-A-E_/TCS and M_L-E-A_/TCS was determined using MTT assay.

A 2% (v/v) solution of rabbit erythrocyte was firstly prepared. Then, a total of 200 µL rRBC solution was incubated with 200 µL of M_L-E-A_/TCS and M_L-A-E_/TCS (with a final TCS concentration of 20 ~ 160 μg/mL) at 37 ºC for 2 h and then centrifuged at 1000 g at 4 ºC for 5 min. The supernatant was transferred to 96-well plates, and the optical density at 570 nm was measured using a Synergy H1 hybrid multi-mode microplate reader (BioTek Instruments, Inc., USA) to monitor the release of hemoglobin. The negative and positive controls were rRBCs in saline and 0.5% Triton X-100, respectively. The release of hemolysis was measured using Equation (3):

$\text{Hemolysis (\%})=\left[ \left( {OD}_{t}-{OD}_{0} \right)/\left( {OD}_{100}-{OD}_{0} \right) \right]\times100\%$ (3)

Where *OD_t_* is the absorption of erythrocyte in tested formulations at the concentration of t, *OD_0_* is the absorption of erythrocyte in saline, and *OD_100_* is the absorption of erythrocyte in 0.5% Triton X-100.

**2. Results**

**2.1 Characterization of copolymers**

The ^1^H NMR spectra of final copolymers were shown in Fig. S4-6. All the chemical shifts were expressed in parts per million (δ) relative to the solvent signal. The ^1^H NMR spectrum (CDCl_3_) of PLA-PEG-PAE (Fig. S4) showed peaks at δ=5.19 ppm (-COCH(CH_3_)O-), δ=4.02 ppm (-COO-CH_2_-CH_2_-), δ= 3.79 ppm and 3.65 ppm (-O-CH_2_-CH_2_-) and (-N-CH_2_-CH_2_-), δ=3.20 ppm (-CO-N-CH_2_-CH_2_-), δ= 2.92 ppm (N-CH_2_-CH_2_-), δ= 2.69 ppm and δ= 2.48 ppm (the proton g and h on Pyridine Ring), δ=1.89 ppm (-CH_2_-COO-CH_2_-), δ= 1.52 ppm (-COO-CH_2_-CH_2_-) and δ= 1.29 ppm (-CH_2_-CH_3_). The ^1^H NMR spectrums (CDCl_3_) of PLA-PAE-mPEG (Fig. S5) and PAE-PLA-mPEG (Fig. S6) also showed the characteristic peaks of PLA block, PAE block and mPEG block, suggesting the two copolymers were obtained.


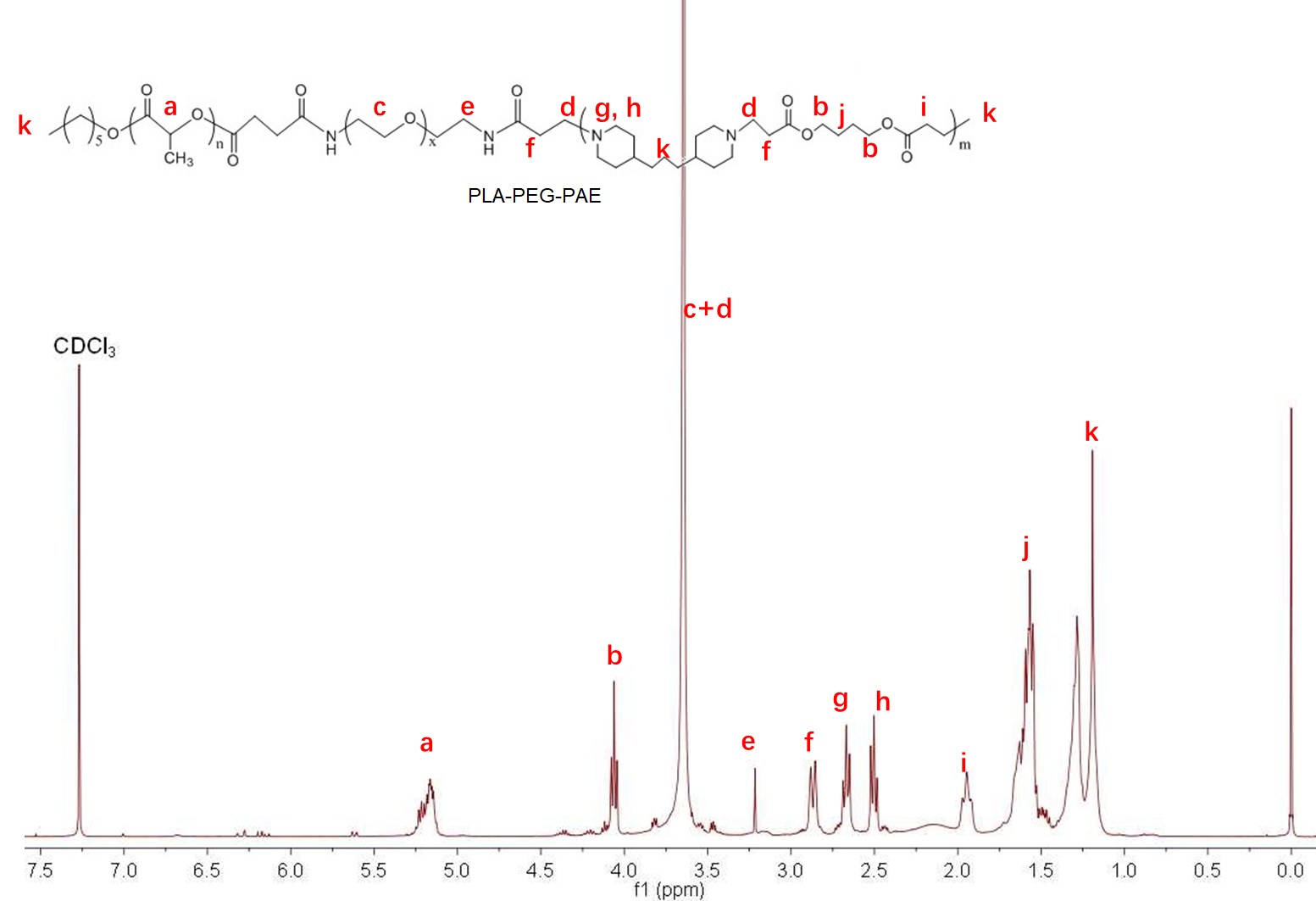


**Fig. S4** ^1^H NMR spectrum of PLA_5K_-PEG_5K_-PAE_5K_


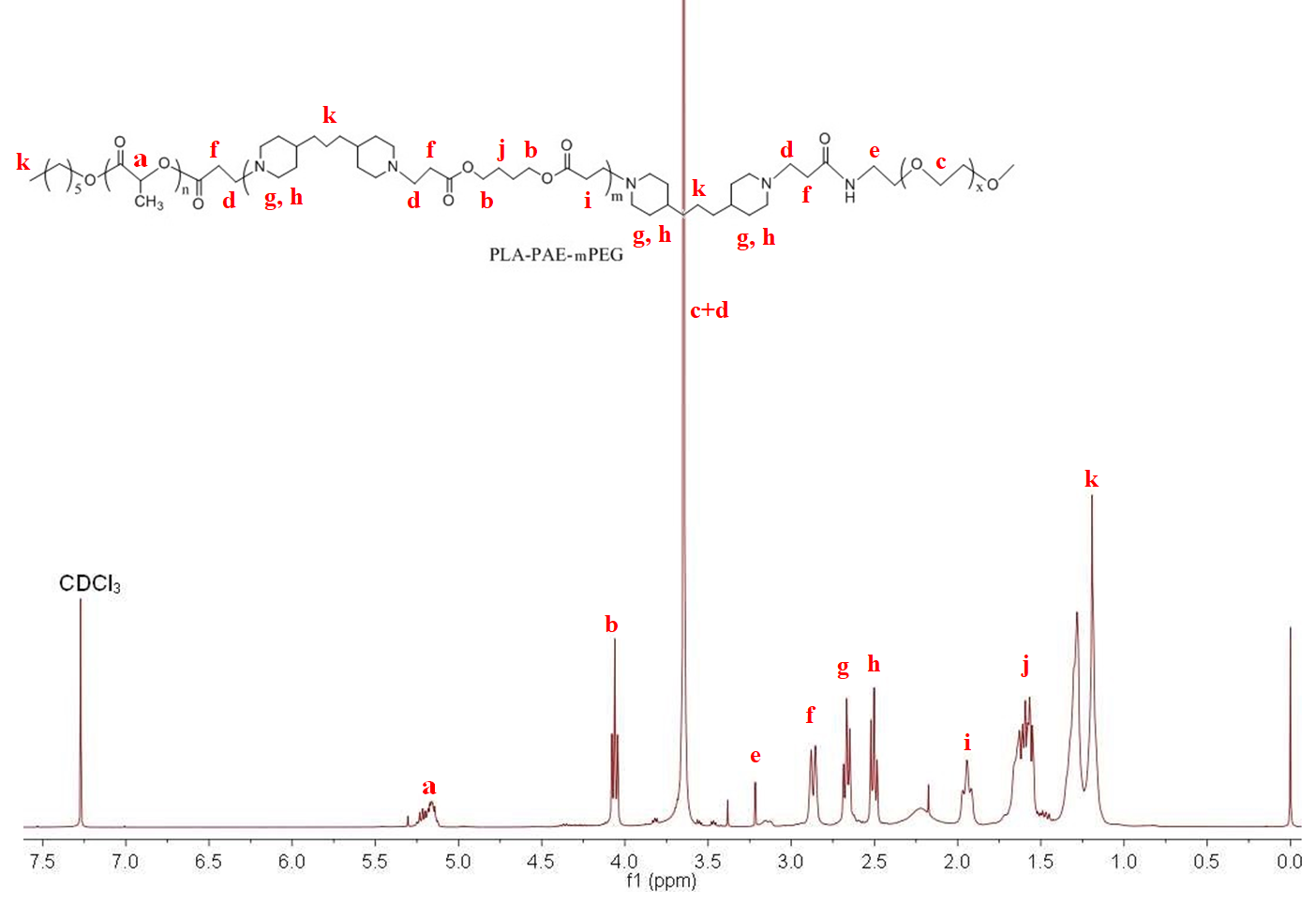


**Fig. S5** ^1^H NMR spectrum of PLA_5K_-PAE_5K_-mPEG_5K_


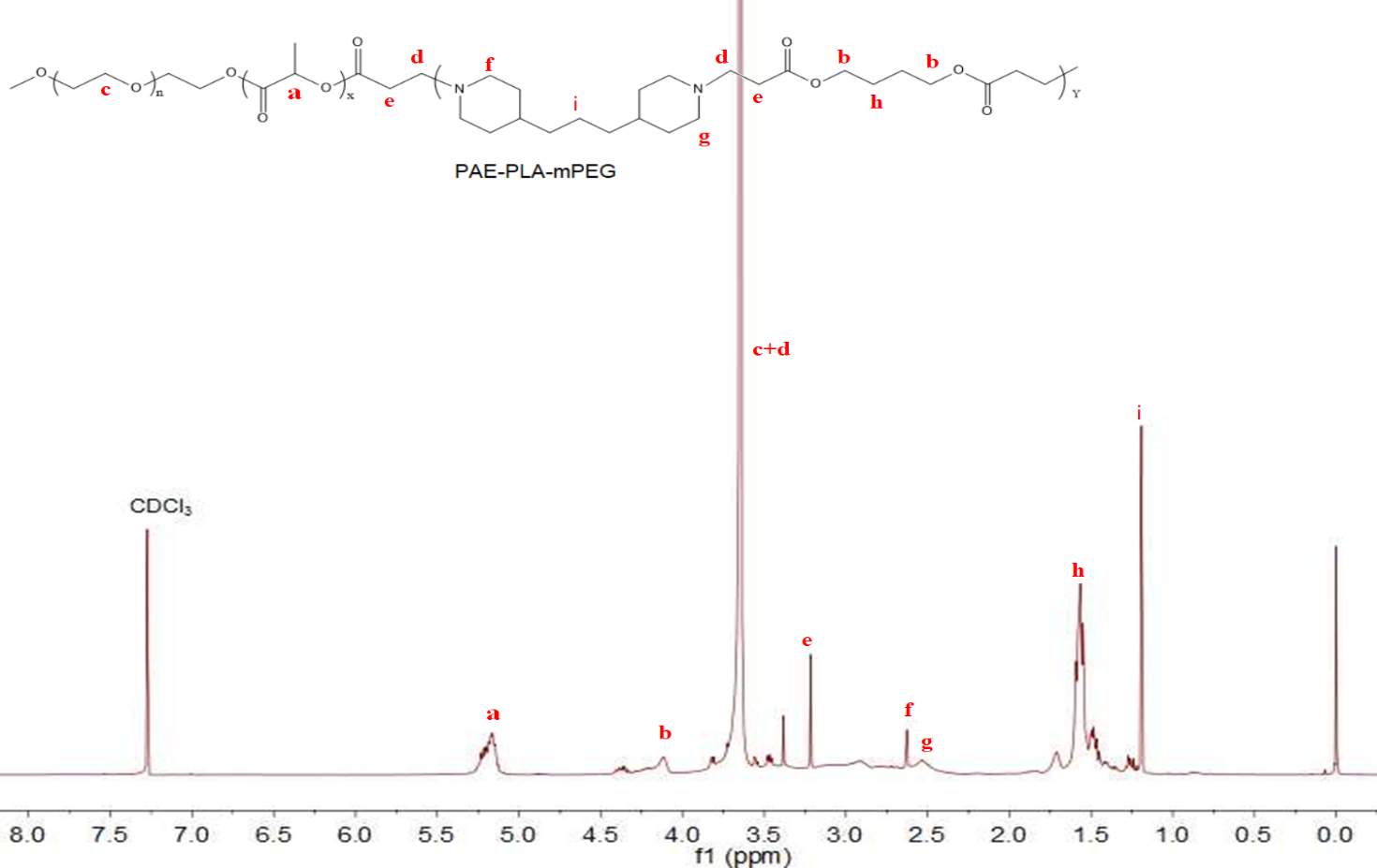


**Fig. S6** ^1^H NMR spectrum of PAE_5K_-PLA_5K_-mPEG_5K_

The composition and characterization of the PLA_5K_-PAE_5K_-mPEG_5K_, PLA_5K_-PEG_5K_-PAE_5K_ and PAE_5K_-PLA_5K_-mPEG_5K_ were presented in Table S1. From the GPC results (Fig. S7), the elution time of PAE-PLA-mPEG, PLA-PAE-mPEG and PLA-PEG-PAE was 6.411 min, 6.539 min and 6.367 min. The number average molecular weight (M_n_) of PAE-PLA-mPEG, PLA-PAE-mPEG and PLA-PEG-PAE was 15008, 14970 and 15016, respectively. Additionally, all the copolymers showed unimodal distribution with a polydispersity around 1.6. Basing on the narrow molecular weight distribution, it was reasonable to assume that most of the copolymers has the triblock structure.

**Table S1** The characteristic of synthesized copolymers

| Copolymer | M_n_ of PLA^a^ (g/mol) | M_n_ of PAE^c^ (g/mol) | M_n_ of PEG (g/mol) | M_n_ of copolymer^b^ (g/mol) | PDI^b^ |
| --- | --- | --- | --- | --- | --- |
| PLA_5K_-PEG_5K_-PAE_5K_ | 4982 | 5034 | 5000 | 15016 | 1.59 |
| PLA_5K_-PAE_5K_-mPEG_5K_ | 4995 | 4975 | 5000 | 14970 | 1.53 |
| PAE_5K_-PLA_5K_-mPEG_5K_ | 5018 | 4990 | 5000 | 15008 | 1.64 |

^a^ M_n_ was calculated from ^1^H NMR.

^b^ M_n_ was determined from GPC

^c^ Theoretical M_n_ of PAE block was calculated as M_n_ copolymer^b^ -M_n_ PEG - M_n_ PLA^a^.

^b^ PDI was determined by GPC.

**
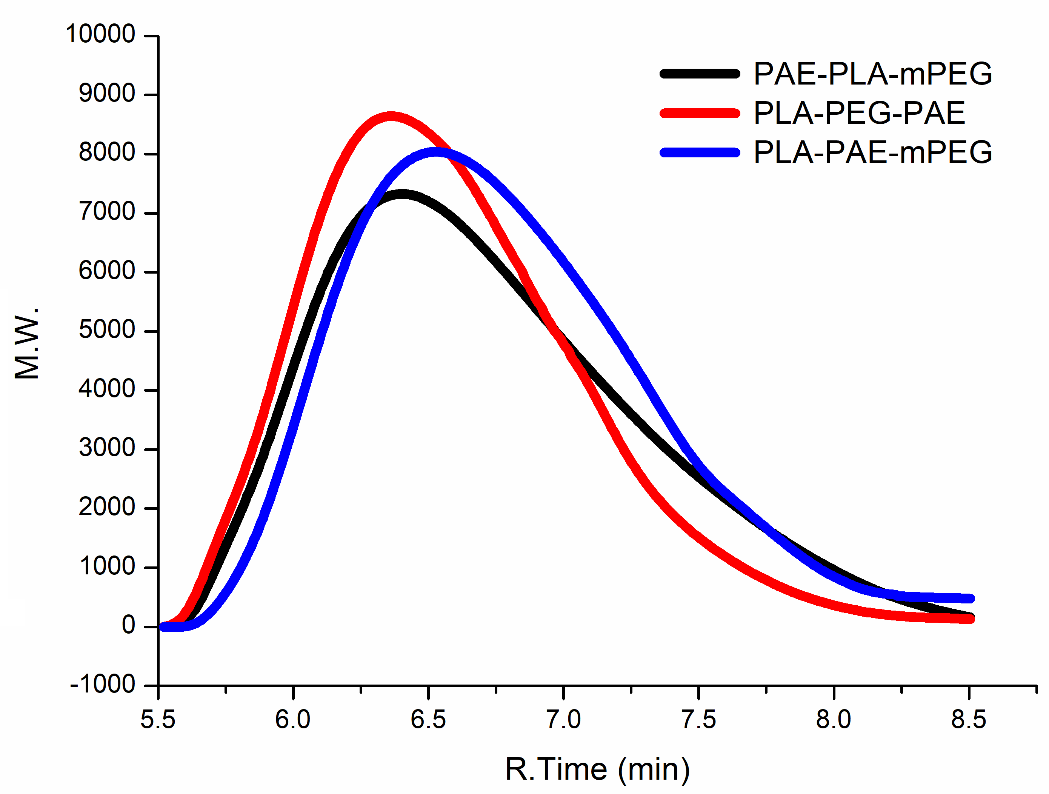
**

**Fig. S7** The typical GPC spectrum of PAE_5K_-PLA_5K_-mPEG_5K_, PLA_5K_-PEG_5K_-PAE_5K_ and PLA_5K_-PAE_5K_-mPEG_5K_

**2.2 *In vitro* cytotoxicity and hemolysis analysis**

*In vitro* cytotoxicity of M_L-E-A_/TCS and M_L-A-E_/TCS against Vero cells was illustrated in Fig. S8A. Both M_L-E-A_/TCS and M_L-A-E_/TCS exhibited limited cytotoxicity. The cell viabilities were all above 85% even at the highest TCS concentration of 160 μg/mL, which was much higher than that used for *in vivo* biofilm eradication evaluation (~30 μg/mL).

During antibacterial treatments *in vivo*, cytotoxicity to blood cells needs to be considered*.* The hemolytic activities of M_L-E-A_/TCS and M_L-A-E_/TCS were further tested. As shown in Fig. S8B, the hemolysis rate increased with the concentration of TCS-loaded micelles. However, the rate change was tolerable (≈10%). No significant hemolytic behaviors were observed even at the highest TCS concentration which was much higher than that used in this study, suggesting that both M_L-E-A_/TCS and M_L-A-E_/TCS used in our study had good hemocompatibility.

**
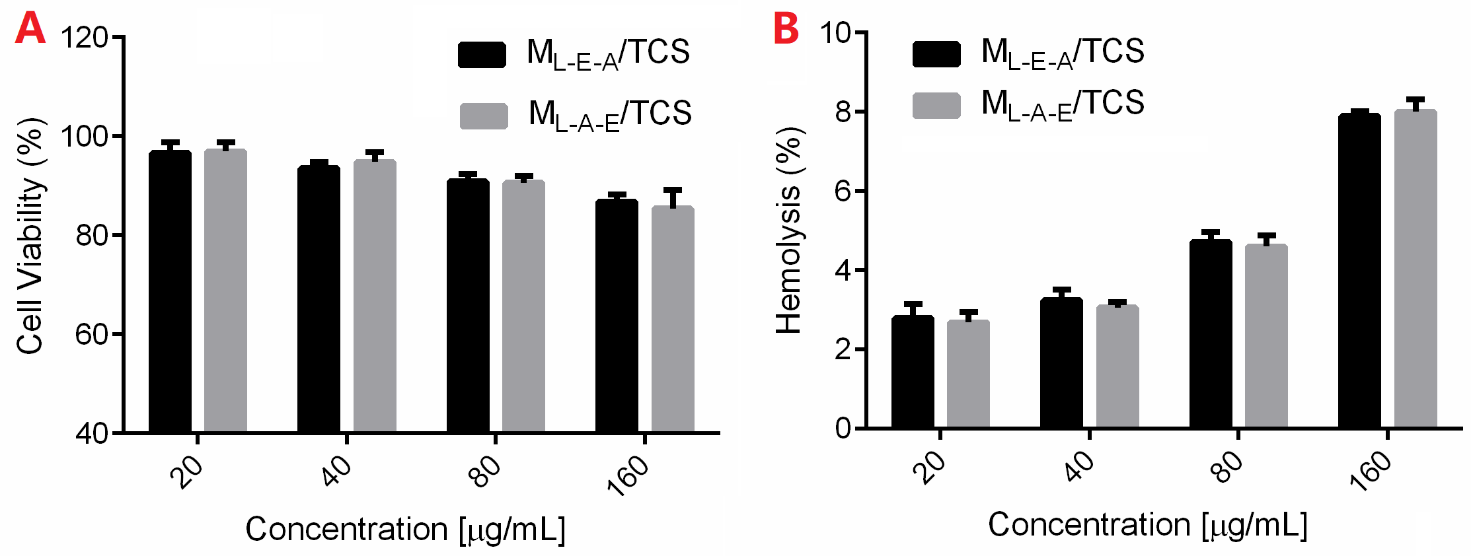
**

**Fig. S8** The *in vitro* cytotoxicity (A) and hemolysis behavior (B) of M_L-E-A_/TCS and M_L-A-E_/TCS as a function of TCS concentration (mean±SD, n=6).
